# A microbial causal mediation analytic tool for health disparity and applications in body mass index

**DOI:** 10.1186/s40168-023-01608-9

**Published:** 2023-07-27

**Authors:** Chan Wang, Jiyoung Ahn, Thaddeus Tarpey, Stella S. Yi, Richard B. Hayes, Huilin Li

**Affiliations:** 1 Department of Population Health, Division of Biostatistics, New York University Grossman School of Medicine, New York, NY 10016 USA; 2 Department of Population Health, Division of Epidemiology, New York University Grossman School of Medicine, New York, NY 10016 USA; 3 Department of Population Health Section for Health Equity, New York University Grossman School of Medicine, New York, 10016 USA

**Keywords:** Casual mediation model, Health disparity, Manipulable disparity measure, Microbiome mediator, Non-manipulable exposure

## Abstract

**Background:**

Emerging evidence suggests the potential mediating role of microbiome in health disparities. However, no analytic framework can be directly used to analyze microbiome as a mediator between health disparity and clinical outcome, due to the non-manipulable nature of the exposure and the unique structure of microbiome data, including high dimensionality, sparsity, and compositionality.

**Methods:**

Considering the modifiable and quantitative features of the microbiome, we propose a microbial causal mediation model framework, SparseMCMM_HD, to uncover the mediating role of microbiome in health disparities, by depicting a plausible path from a non-manipulable exposure (e.g., ethnicity or region) to the outcome through the microbiome. The proposed SparseMCMM_HD rigorously defines and quantifies the manipulable disparity measure that would be eliminated by equalizing microbiome profiles between comparison and reference groups and innovatively and successfully extends the existing microbial mediation methods, which are originally proposed under potential outcome or counterfactual outcome study design, to address health disparities.

**Results:**

Through three body mass index (BMI) studies selected from the curatedMetagenomicData 3.4.2 package and the American gut project: China vs. USA, China vs. UK, and Asian or Pacific Islander (API) vs. Caucasian, we exhibit the utility of the proposed SparseMCMM_HD framework for investigating the microbiome’s contributions in health disparities. Specifically, BMI exhibits disparities and microbial community diversities are significantly distinctive between reference and comparison groups in all three applications. By employing SparseMCMM_HD, we illustrate that microbiome plays a crucial role in explaining the disparities in BMI between ethnicities or regions. 20.63%, 33.09%, and 25.71% of the overall disparity in BMI in China-USA, China-UK, and API-Caucasian comparisons, respectively, would be eliminated if the between-group microbiome profiles were equalized; and 15, 18, and 16 species are identified to play the mediating role respectively.

**Conclusions:**

The proposed SparseMCMM_HD is an effective and validated tool to elucidate the mediating role of microbiome in health disparity. Three BMI applications shed light on the utility of microbiome in reducing BMI disparity by manipulating microbial profiles.

Video Abstract

**Supplementary Information:**

The online version contains supplementary material available at 10.1186/s40168-023-01608-9.

## Background

Health disparities refer to the inequalities in the quality of health, health care, and health outcomes experienced by groups that are usually classified by race, ethnicity, and region. Many factors, including genetics, social-economic status, culture, dietary habits, and geographical conditions, contribute to health disparities between groups. Researchers have long been interested in identifying the modifiable environmental determinants of health disparity to pave the way to improve health equity. However, environmental exposures are often numerous, ubiquitous, descriptive, or hard to measure, which makes this task difficult.

**Fig. 1:**
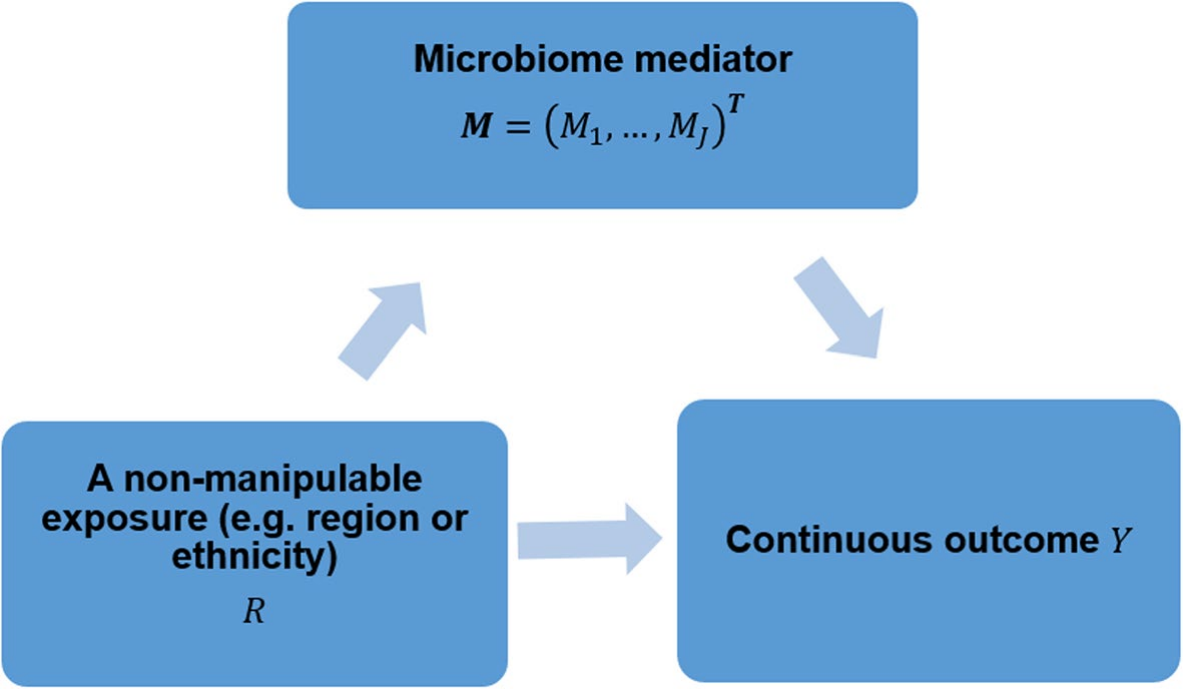
Microbiome (*M*) may play a mediating role in the health disparity of the continuous outcome (*Y*) between two categories of a non-manipulable exposure group (e.g., region or ethnicity) (*R*). We aim to investigate how much disparity of the outcome *Y* can be reduced by manipulating microbiome profiles

The gut microbiome is the aggregate of all genomes harbored by gut microbiota, which is the collection of all microbes that reside in the human gut. Benefiting from the advent of high throughput sequencing technologies, a great number of microbiome studies have been conducted to quantitatively characterize microbiota and understand its role in human health [1–4]. On the one hand, the gut microbiome has been closely linked with host metabolic, immune, and neuroendocrine functions [5–12]. On the other hand, many environmental and social factors, such as diet, drugs, lifestyle, psychological state, and behavior, aid in shaping gut microbial profiles [13–16]. Recently, the mediating role of the microbiome between these environmental exposures and various human diseases, including obesity, type 2 diabetes, inflammatory bowel disease, depression, and different cancers, has been investigated and recognized [17–22]. Given the modifiable and quantitative features of the microbiome, we here aim to disentangle health disparities by quantifying the extent of the observed disparity in outcome that could be reduced if the gut microbial profile was modified. Figure 1 depicts a schematic mediation framework to answer such questions. Here, the disparity group, e.g., ethnicity or region, is the exposure denoted by *R*; the gut microbial profile is the mediator denoted by *M*; and the continuous study outcome, e.g., body mass index (BMI), is denoted by *Y*.

There are several existing mediation analysis frameworks tailored for non-manipulable exposures, such as ethnicity, region, sex, or socioeconomic position [23]; however, due to the unique structure of microbiome data, including the high dimensionality, sparsity, and compositionality, these approaches are not immediately applicable for analyzing the microbiome as the mediator for the study of health disparities. Recently, we developed a rigorous Sparse Microbial Causal Mediation Model (SparseMCMM) [12] for interrogating the mediating role of microbiome in a typical three-factor (randomized treatments, microbiome as mediator, and outcome) clinical trial causal study design. SparseMCMM quantifies the overall mediation effect of microbiome community and the component-wise mediation effect for each individual microbe under the counterfactual framework, identifies the signature causal microbes with regularization strategies, and tests the mediation effects while fully acknowledging the unique structure of microbiome data. In this paper, we extend SparseMCMM to a non-manipulable exposure setting, propose a microbial causal mediation framework for health disparity study, and denote it as SparseMCMM_HD (SparseMCMM for Health Disparity). As VanderWeele and Robinson [24] discussed, causal interpretation of a non-manipulable exposure, i.e., ethnicity or region, is not definable in the traditional counterfactual framework, because a hypothetical intervention on a non-manipulable exposure is not possible. Instead, one can interpret the causality of health inequality by the hypothesized intervention effect on the manipulable mediating variable. Thus, in SparseMCMM_HD, we aim to quantify the overall health inequality on the outcome (called overall disparity), the health inequality effect that would be eliminated by equalizing microbiome profiles across ethnic or regional groups (called manipulable disparity), and the healthy inequality effect that would remain even after microbiome profiles across ethnic or regional groups were equalized (called residual disparity). In addition, we equip two hypothesis tests to examine the mediating role of microbiome in health disparity and statistically identify which specific microbes contribute to it.

Obesity (defined via BMI) is a global epidemic and a persistent public health problem [25]. It is well documented that the prevalence of adult obesity is distributed unevenly across ethnic groups and regions. Partial effect of the manipulable exposures such as diet, medication, and antibiotics use [17–19] on obesity has been shown to be mediated through microbiome. In addition, accumulating evidence indicates that the gut microbial profile varies across ethnicities as well as geographically [23, 26, 27]. Together, these studies suggest that the microbiome may play a mediating role in the ethnic or regional disparity of obesity. It is crucial to investigate rigorously how much health inequalities in BMI can be reduced by manipulating microbiome profiles. Utilizing SparseMCMM_HD, we investigate the role of the microbiome in the disparity of BMI between ethnicities and regions. We use the curated microbiome data from the curatedMetagenomicData 3.4.2 package [28] and the American Gut Project (AGP) (www.americangut.org) to illustrate a clear and plausible causal path analysis to understand the current ethnic or regional disparity in BMI and identify a comprehensive set of mediating microbial taxa. The proposed analytic pipeline is available through an interactive web app at https://chanw0.shinyapps.io/sparsemcmm_hd/. We believe this novel pipeline will be useful for investigating the manipulable disparity through gut microbiome and understanding the causes of the health disparity.

## Methods

### SparseMCMM_HD framework

#### Casual mediation model

Suppose there are $I$ subjects from two categories of a non-manipulable exposure group (e.g., ethnicity or region), $J$ taxa, and $K$ covariates. Subscripts $i$, $j$, and $k$, indicate a subject, a taxon, and a covariate, respectively. For the $i$ th subject, let ${R}_{i}=1$ or 0 indicate the reference or comparison group, let ${{\varvec{M}}}_{i}=({M}_{i1},\dots ,{M}_{iJ}{)}^{T}$ be the microbiome relative abundance vector with the constraint $\sum_{j=1}^{J}{M}_{ij}=1$, and let ${{\varvec{X}}}_{i}=({X}_{i1},\dots ,{X}_{iK}{)}^{T}$ represent the covariates, and let ${Y}_{i}$ be a continuous outcome of interest.

To statistically describe the causal relationships shown in Fig. 1, following our previous work [12], we use the linear log-contrast model to regress the continuous outcome on the non-manipulable exposure, microbiome compositions, and interactions between the non-manipulable exposure and microbiome compositions, while adjusting the confounding covariates:

1 $$\begin{array}{cc}{Y}_{i}=& {\alpha }_{0}+{{\varvec{\upalpha}}}_{X}^{T}{{\varvec{X}}}_{i}+{\alpha }_{R}{R}_{i}+{{\varvec{\upalpha}}}_{M}^{T}[{\text{log}}({{\varvec{M}}}_{i})]+{{\varvec{\upalpha}}}_{C}^{T}[{\text{log}}({{\varvec{M}}}_{i})]{R}_{i}+{\epsilon }_{i},\\ & \text{subject }\text{to }{{\varvec{\upalpha}}}_{M}^{T}1=0,\text{and }{{\varvec{\upalpha}}}_{C}^{T}1=0,\end{array}$$

where ${\alpha }_{0}$ is the intercept, ${\alpha }_{R}$ is the coefficient of the non-manipulable exposure, ${{\varvec{\upalpha}}}_{X}=({\alpha }_{X1},\dots ,{\alpha }_{XK}{)}^{T}$, ${{\varvec{\upalpha}}}_{M}=({\alpha }_{M1},\dots ,{\alpha }_{MJ}{)}^{T}$, and ${{\varvec{\upalpha}}}_{C}=({\alpha }_{C1},\dots ,{\alpha }_{CJ}{)}^{T}$ are the vectors of coefficients of covariates, microbiome compositions, and interactions between the non-manipulable exposure and microbiome compositions, respectively. Due to the compositionality of the microbiome data $\sum_{j=1}^{J}{M}_{ij}=1$, ${{\varvec{\upalpha}}}_{M}$ and ${{\varvec{\upalpha}}}_{C}$ are additionally subject to ${{\varvec{\upalpha}}}_{M}^{T}1=0, \text{and }{{\varvec{\upalpha}}}_{C}^{T}1=0$. ${\epsilon }_{i}\sim N(0,{\sigma }^{2})$ is the error term. On the other hand, the Dirichlet regression [29] is used to model the microbial relative abundance as a function of the non-manipulable exposure and covariates:

2 $$\begin{array}{cc}E\left[{M}_{ij}\right]=& \frac{{\gamma }_{j}\left({R}_{i}, {{\varvec{X}}}_{i}\right)}{\sum_{m=1}^{J}{\gamma }_{m}\left({R}_{i},{{\varvec{X}}}_{i}\right)},\\ {\text{log}}\left\{{\gamma }_{j}\left({R}_{i},{{\varvec{X}}}_{i}\right)\right\}=& {\beta }_{0j}+{\beta }_{Rj}{R}_{i}+{{\varvec{\upbeta}}}_{Xj}^{T}{{\varvec{X}}}_{i}.\end{array}$$

Specifically, we assume that ${{\varvec{M}}}_{i}|({R}_{i},{{\varvec{X}}}_{i})\sim {\text{Dirichlet}}\left({\gamma }_{1}({R}_{i},{{\varvec{X}}}_{i}),\dots ,{\gamma }_{J}({R}_{i},{{\varvec{X}}}_{i})\right)$, and their microbial relative means are linked with the non-manipulable exposure and covariates (${R}_{i},{{\varvec{X}}}_{i}$) in the generalized linear model fashion with a log link. ${\beta }_{0j}$ is the intercept and ${\beta }_{Rj}$ and ${{\varvec{\beta}}}_{Xj}$ are the coefficients of the non-manipulable exposure and covariates for the $j$ th taxon, respectively.

#### Definition of disparity measures in the counterfactual framework

As discussed in the “Background” section, we propose to conceptualize an overall disparity measure (ODM) on the outcome that can be decomposed into manipulable disparity measure (MDM) and residual disparity measure (RDM). MDM represents the portion of disparity that would be eliminated by equalizing microbiome profiles between comparison and reference groups, and RDM represents the portion that would remain even after microbiome profiles between comparison and reference groups were equalized. With the counterfactual notation, mathematically we have:

$$\begin{array}{c}\mathrm{ODM}=\mathrm{MDM}+\mathrm{RDM},\\ \mathrm{MDM}=E\left[E[{Y}_{{{\varvec{M}}}_{{\varvec{x}}}(1)}|R=1,{\varvec{x}}]\right]-E\left[E\left[{Y}_{{{\varvec{M}}}_{{\varvec{x}}}\left(0\right)}|R=1,{\varvec{x}}\right]\right], \mathrm{and }\\ \mathrm{RDM}=E\left[E[{Y}_{{{\varvec{M}}}_{{\varvec{x}}}(0)}|R=1,{\varvec{x}}]-E[{Y}_{{{\varvec{M}}}_{{\varvec{x}}}(0)}|R=0,{\varvec{x}}]\right].\end{array}$$

Here, ${{\varvec{M}}}_{{\varvec{x}}}(0)$ (${{\varvec{M}}}_{{\varvec{x}}}(1)$) is a random value from the microbiome distribution of the reference (comparison) population with given covariates ${\varvec{x}}$. ${Y}_{{\varvec{m}}}$ denotes an individual’s potential counterfactual outcome if his or her microbial mediators were set to ${\varvec{m}}$, where ${\varvec{m}}$ can be ${{\varvec{M}}}_{{\varvec{x}}}(0)$ or ${{\varvec{M}}}_{{\varvec{x}}}(1)$. $E[{Y}_{{{\varvec{M}}}_{{\varvec{x}}}(0)}|R=0,{\varvec{x}}]$ ($E[{Y}_{{{\varvec{M}}}_{{\varvec{x}}}(1)}|R=1,{\varvec{x}}]$) denotes the expected outcome for a reference (comparison) individual with given covariates ${\varvec{x}}$, $E\left[{Y}_{{{\varvec{M}}}_{{\varvec{x}}}\left(0\right)}|R=1,{\varvec{x}}\right]$ denotes the expected outcome for a comparison individual with given covariates ${\varvec{x}}$ if their microbial mediators were set to a random value from that of the reference population with the same covariates ${\varvec{x}}.$

#### MDM, RDM, and ODM expressions

Two assumptions must be satisfied for the identification of MDM, RDM, and ODM [24, 30]. The effect of the non-manipulable exposure *R* on the outcome *Y* are unconfounded conditional on all covariates ${\varvec{X}}$, i.e., $Y\coprod R|{\varvec{X}}$ and the effects of the mediator ***M*** on the outcome *Y* are unconfounded conditional on the non-manipulable exposure *R* and all covariates ${\varvec{X}}$, i.e., $Y\coprod {\varvec{M}}|R,{\varvec{X}}$. With these sufficient identifiability assumptions and the models (1)-(2) proposed in the SparseMCMM_HD framework, disparity measures MDM, RDM, and ODM can be further expressed, respectively, as follows (see Section S1 for the detailed derivations):

$$\begin{array}{c}\mathrm{MDM}=\sum_{j=1}^{J}({\alpha }_{Mj}+{\alpha }_{Cj})\left\{E\left[\mathrm{log}\left({M}_{j}\right)|R=1,{\varvec{x}}\right]-E\left[\mathrm{log}\left({M}_{j}\right)|R=0,{\varvec{x}}\right]\right\},\\ \mathrm{RDM}= {\alpha }_{R}+{{\varvec{\upalpha}}}_{C}^{T}E\left[{\text{log}}\left({\varvec{M}}\right)|R=0,{\varvec{x}}\right]={\alpha }_{R}+\sum_{j=1}^{J}{\alpha }_{Cj}E\left[\mathrm{log}\left({M}_{j}\right)|R=0,{\varvec{x}}\right],\end{array}$$

and

$$\begin{array}{cc}\mathrm{ODM}& =\mathrm{MDM}+\mathrm{RDM}\\ & ={\alpha }_{R}+\sum_{j=1}^{J}({\alpha }_{Mj}+{\alpha }_{Cj})E\left[\mathrm{log}\left({M}_{j}\right)|R=1,{\varvec{x}}\right]-\sum_{j=1}^{J}{\alpha }_{Mj}E\left[\mathrm{log}\left({M}_{j}\right)|R=0,{\varvec{x}}\right],\end{array}$$

where $E\left[\mathrm{log}\left({M}_{j}\right)|R=r,{\varvec{x}}\right]=\uppsi \left[{\gamma }_{j}\left(R=r,{\varvec{x}}\right)\right]-\uppsi \left[{\sum }_{m=1}^{J}{\gamma }_{m}\left(R=r,{\varvec{x}}\right)\right]$, ${\gamma }_{j}\left(R=r,{\varvec{x}}\right)=\mathrm{exp}\left({\beta }_{0j}+{\beta }_{Rj}r+{{\varvec{\upbeta}}}_{Xj}^{T}{\varvec{x}}\right)$, $r=0$ or 1, and $\uppsi \left(\bullet \right)=\frac{d}{dx}\mathrm{ln}\left(\Gamma \left(x\right)\right)$ is the digamma function, with given covariates ${\varvec{x}}.$

Note that these mathematical expressions of RDM and MDM are the same as the formulas of causal direct effect of treatment and mediation effect through microbiome correspondingly on the outcome in the typical three-factor causal design based on the traditional causal mediation inference, developed in our SparseMCMM [12]. Analogous to ME in SparseMCMM, MDM is the summation of individual mediation effects from each taxon ${MDM}_{j}$: $\mathrm{MDM }:=\sum_{j=1}^{J}{MDM}_{j}$ and ${MDM}_{j}=({\alpha }_{Mj}+{\alpha }_{Cj})\{E[\mathrm{log}({M}_{j})|R=1,{\varvec{x}}]-E[\mathrm{log}({M}_{j})|R=0,{\varvec{x}}]\}$. ${MDM}_{j}$ thus is non-zero only when both the *j*th microbial effect on the outcome and the exposure effect on the *j*th taxon are not zero. Therefore, SparseMCMM_HD illuminates the mediating role of microbiome in the health disparity of outcome and quantifies the manipulable disparity for overall microbiome community and for each specific taxon, respectively.

#### Parameter estimation

Analogous to SparseMCMM [12], we employ a two-step procedure to estimate the regression parameters in models (1)–(2) to obtain the estimated RDM, MDM, and ${MDM}_{j}$ for each taxon, and ODM. Furthermore, SparseMCMM_HD has the full capability to perform variable selection to select the signature causal microbes that play mediating roles in the disparity of the continuous outcome with regularization strategies. Specifically, L_1_ norm and group-lasso penalties are incorporated for variable selection. To account for the biases introduced by the regularization techniques employed, we further implement splitting strategy [31], which can handle arbitrary penalties and provide asymptotically validated inference. We also incorporate this splitting strategy in the SparseMCMM package to refine its estimation procedure.

#### Hypothesis tests for manipulable disparity

Similarly, we employ the hypothesis tests for mediation effects in SparseMCMM to examine whether the microbiome has any mediation effect on the disparity in the outcome, at the community and taxon levels, respectively. Specifically, regarding the null hypothesis of no manipulable disparity ${H}_{0}:\mathrm{MDM}=0$, the first test statistic is defined as OMD = $\widehat{MDM}$, the estimator of the manipulable disparity. OMD examines whether or not the whole microbiome plays a mediating role in health disparities. Meanwhile, we consider another null hypothesis, ${H}_{0}:{MDM}_{j}=0, \forall j\in \left\{1,\cdots ,J\right\}$ and define the second test statistic as CMD = $\sum_{j=1}^{J}{\widehat{MDM}}_{j}^{2}$, the summation of squared estimators of individual mediation effects across all taxa. CMD examines whether or not at least one taxon mediates the health disparities. Permutation procedure is employed to assess the significance of these two test statistics. This provides a mechanism to check whether the microbiome has any impact on health disparity that could be potentially eliminated through the microbiome.

### Control for confounding covariates

Due to the non-manipulable nature of the exposure in health disparity research, in principle, it is not possible to design a randomized trial on the exposure of interest to eliminate the potential confounding effect on the interested causal pathway. Many studies on health disparity are observational and usually include significant degrees of confounding, due to factors such as lifestyle, health status, and disease history. We want to emphasize that it is a necessary step to control for confounding covariates before utilizing the proposed SparseMCMM_HD to estimate RDM, MDM, and ODM in a typical observational study. Specifically, we propose to perform propensity score matching (PSM) [32], which is a commonly used method in biomedical research to create a balanced covariate distribution between two groups, to control confounding covariates in our applications (see Section S2). Standardized mean difference (SMD) is used to evaluate the balance of the covariate distributions between groups. An SMD that is less than 0.1 indicates a balanced distribution [33]. The matched data will then be used to quantify RDM, MDM, and ODM and examine whether the microbiome could reduce the health disparity between two non-manipulable exposure groups. Note that the PSM procedure controlling for confounding covariates has been included as a preprocessing step in the proposed SparseMCMM_HD analytic pipeline.

### curatedMetagenomicDataV3.4.2

The curatedMetagenomicData 3.4.2 package [28] provides a curated human microbiome meta dataset aggregated from 86 shotgun sequencing cohorts in 6 body sites. The raw sequencing data were processed using the same bioinformatics protocol and pipelines. Each sample has 6 types of data available including gene family, marker abundance, marker presence, pathway abundance, pathway coverage, and taxonomic (relative) abundance. The taxonomic abundance was calculated with MetaPhlAn3, and metabolic functional potential was calculated with HUMAnN3. The manually curated clinical and phenotypic metadata are available as well. More details can be found in the curatedMetagenomicData package document [28]. Here, we focus on the healthy subjects to explore the relationship among region, microbiome, and BMI. Specifically, we chose the subjects from all cohorts based on the following inclusion criteria: (1) healthy status; (2) no missing values in BMI, gender, and age; (3) age $\ge$ 18; (4) no pregnant; (5) currently no antibiotic use; (6) currently no alcohol consumption; (7) no smoking; and (8) fecal sample with more than 1250 sample reads. In addition, when multiple samples available for a subject, we randomly selected one sample. Overall, we identified 4868 healthy adults from various regions. Here, we further focus on three regional groups which have large sample sizes: China (*n* = 570), United States (USA; *n* = 350), and United Kingdom (UK; *n* = 1019) for the analysis in the main text. Specifically, we conducted two comparison studies: China-USA and China-UK comparisons to investigate the regional difference of BMI in the China group compared to the USA and UK groups, respectively.

### American gut project

The AGP project is a crowd-sourcing citizen science cohort to describe the comprehensive characterization of human gut microbiota and to identify factors being linked to human microbiota. The AGP includes 16S rRNA V4 gene sequences from more than 8000 fecal samples using standard pipelines and the host metadata. Detailed descriptions can be found in Liu et al. and Hu et al. [1, 34]. Our primary investigation is on the disparity of BMI between Asian or Pacific Islander (API) and non-Hispanic Caucasian adults. We selected a subset of the AGP data based on the following inclusion criteria: (1) USA resident; (2) Asian or Pacific Islander or Caucasian ethnicity; (3) no missing values in gender, age, and BMI; (4) age $\ge$ 18; (5) 80 $\ge$ BMI; (6) 210 cm $\ge$ height $\ge$ 80 cm; (7) 200 kg $\ge$ weight $\ge$ 35 kg; (8) fecal sample with more than 1250 sample reads; (9) not duplicate sample; and (10) no self-reported history of inflammatory bowel disease, diabetes, or antibiotic use in the past year. The subjects are filtered out when the reported BMIs are not consistent with the calculated BMI based on the reported heights and weights, i.e., ($\left|{\mathrm{BMI}}_{\mathrm{reported}}-{\mathrm{BMI}}_{\mathrm{calculated}}\right|/{\mathrm{BMI}}_{\mathrm{calculated}}>5\%$). A dataset with 130 API and 2263 Caucasian adults then is used in this paper (Figure S1a).

### Statistical analysis

Data pre-processing and PSM were conducted in three BMI studies. Specifically, for the China-USA and China-UK comparisons, we performed PSM with the parameters described in Section S2 to control for age and gender, with gender being used for exact matching. For the API-Caucasian comparison, as the AGP includes more than 400 covariates that were collected through self-reported surveys, we first implemented several pre-processing steps to prepare the self-reported covariates for the subsequent analysis, including cleaning up the inconsistent definition of variables, and collapsing the sparse categorical variables into fewer and less sparse categories. Details are provided in Section S3. Forty-four covariates were retained for PSM. We performed univariate linear regressions to identify the potential confounding variables for the relationship among ethnicity, microbiome, and BMI. Twenty-three covariates (*p* value $\le$ 0.05; Figure S1b) were identified as confounders that need to be controlled further based on PSM.

With the matched data, alpha (Observed, Shannon, and Simpson indices), and beta diversities (Bray–Curtis dissimilarity and Jensen–Shannon divergence) were used to estimate microbial community-level diversity. *T* tests were used for group comparisons of BMI and alpha diversity. Permutational multivariate analysis of variance (PERMANOVA) [35] was used to assess group difference of beta diversity. We performed the proposed SparseMCMM_HD framework at the species rank (Section S4) to quantify RDM, MDM, and ODM and examine whether the microbiome could explain the health disparity between two non-manipulable exposure groups. The proposed SparseMCMM_HD pipeline was implemented through an interactive web app (https://chanw0.shinyapps.io/sparsemcmm_hd/) for easy exploration. In terms of the splitting strategy used for bias correction in parameter estimation, aligning with discussions on inference-prediction trade-off [31] and data-splitting rules [36], we randomly divided the dataset into two equal halves: the first half is utilized for variable selection, while the second half is dedicated to parameter estimation. The estimates of RDM, MDM, and ${MDM}_{j}$ were then calculated. We repeated this data splitting procedure 50 times to ensure robustness and accuracy in our estimations and inference. The average RDM, MDM, and ${MDM}_{j}$ estimates, and their standard errors and 95% confidence interval (CI) estimates based on 50 times of repetitions were reported. Regarding hypothesis testing, as discussed in [37], we first applied a permutation strategy to the entire dataset. Subsequently, we implemented a data split strategy on the permuted dataset to yield corresponding estimates. The statistical significance of OMD and CMD was established based on the *p* values, which were calculated from 1000 permutations. A *p* value $\le$ 0.05 was considered as statistical significance.

## Results

### Results for curatedMetagenomicDataV3.4.2

#### Matched datasets

**Fig. 2:**
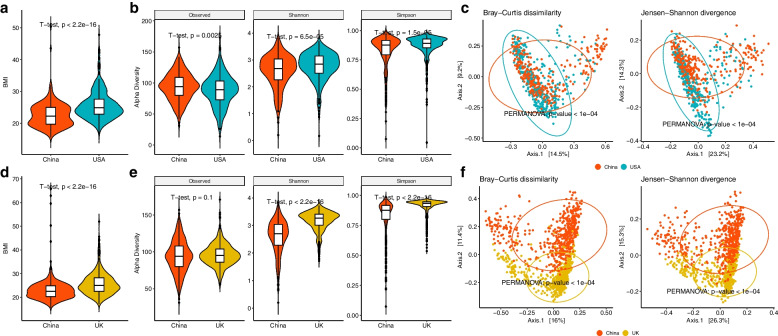
Association analyses in two matched datasets from the curatedMetagenomicData package [28]. **a** Violin plots of BMI in matched Chinese vs. USA subjects. **b** Violin plots of alpha diversities (Observed, Shannon, and Simpson indices) in matched Chinese vs. USA samples. **c** PCoA plots using Bray–Curtis dissimilarity and Jensen–Shannon divergence in matched Chinese and USA samples. **d** Violin plots of BMI in matched Chinese vs. UK subjects. **e** Violin plots of alpha diversities (Observed, Shannon, and Simpson indices) in matched Chinese and UK samples. **f** PCoA plots using Bray–Curtis dissimilarity and Jensen–Shannon divergence in matched Chinese vs. UK samples

With the healthy adults included in the China-USA and China-UK comparisons, by performing the PSM as described in the “Statistical analysis” subsection, we identified 328 matched Chinese-USA subject pairs, and 559 matched Chinese-UK subject pairs, separately. Figures S2 and S3 show that both matched datasets have comparable propensity scores. The SMDs decrease dramatically on the matched subjects (SMD = 0.036 and 0.033), from using all subjects (SMD = 0.302 and 0.470) in both China-USA and China-UK datasets. This indicates that PSM has effectively evened the distribution of confounders between two exposure groups in our studies and practically eliminated or controlled the influence of the confounders. In the well-matched datasets, the China group still has significantly lower average BMIs compared to the matched USA (mean [standard deviation]: 22.64 [3.77] vs. 25.77 [4.56]) and the matched UK (22.98 [4.48] vs. 25.77 [4.79]) groups (Fig. 2a and d).

#### Community level results

The Chinese group has distinctive microbial community diversities, compared to the matched USA or UK group. For alpha diversity, samples from China have lower Shannon and Simpson diversities and a higher observed diversity than the matched USA or UK samples (Fig. 2b and e). For beta diversity, Bray–Curtis dissimilarity and Jensen-Shannon divergence both indicate that the Chinese group is significantly different in community structure from the matched USA or UK groups (PERMANOVA [35] all *p* values < $1.0 \times {10}^{-4}$, Fig. 2c and f).

#### Taxon-level analysis

**Fig. 3:**
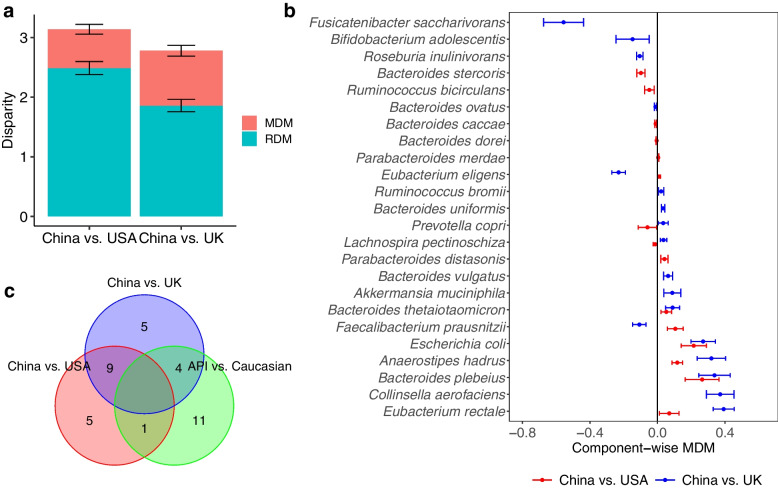
Health disparity analyses in two matched datasets from the curatedMetagenomicData package [28]. **a** The average and standard error of manipulable disparity measure (MDM) and residual disparity measure (RDM) of BMI in the China-USA comparison and China-UK comparison, respectively, based on the splitting strategy with 50 times of repetitions. **b** Component-wise point and 95% CI estimates of ${MDM}_{j}$ for the identified species that have mediation effects on the differences of BMI between matched Chinese vs. USA subjects and between matched Chinese vs. UK subjects, respectively. 95% CI estimates of ${MDM}_{j}$ were calculated based on the splitting strategy with 50 times of repetitions. **c** Venn diagram to show the relationship of the species playing mediation effects in the disparity of BMI among China-USA, China-UK, and API-Caucasian comparisons. API Asian or Pacific Islander

After implementing the filtering criteria described in Section S4, 25 species remained in both matched datasets (China vs. USA and China vs. UK). The testing results for OMD and CMD show that the overall and component-wise MDMs through microbiome are significant in both data sets for regional differences in BMI (all *p* values < 0.001 based on 1000 permutations). Figure 3a shows that the average ODM of BMI are 3.15 and 2.78, respectively, for the matched Chinese and USA subjects, and the matched Chinese and UK subjects; the corresponding average MDMs due to microbiome are 0.65 and 0.92. These results suggest that 20.63% and 33.09% of the disparity in BMI between the Chinese and matched USA and UK groups, respectively, would be eliminated if the between-group microbiome profiles were equalized.

**Fig. 4:**
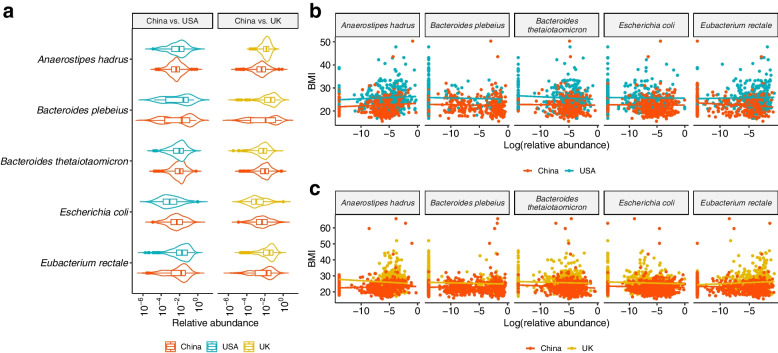
Five species who play positive mediation roles in the disparity of BMI in both China-USA and China-UK comparisons. **a** Violin plots illustrating the relative abundances of these five identified species in the matched Chinese and USA samples, and the matched Chinese and UK samples, respectively. **b** Scatterplots of BMI and the relative abundances of these five identified species in the matched Chinese and USA subjects, and the matched Chinese and UK subjects, respectively

**Fig. 5:**
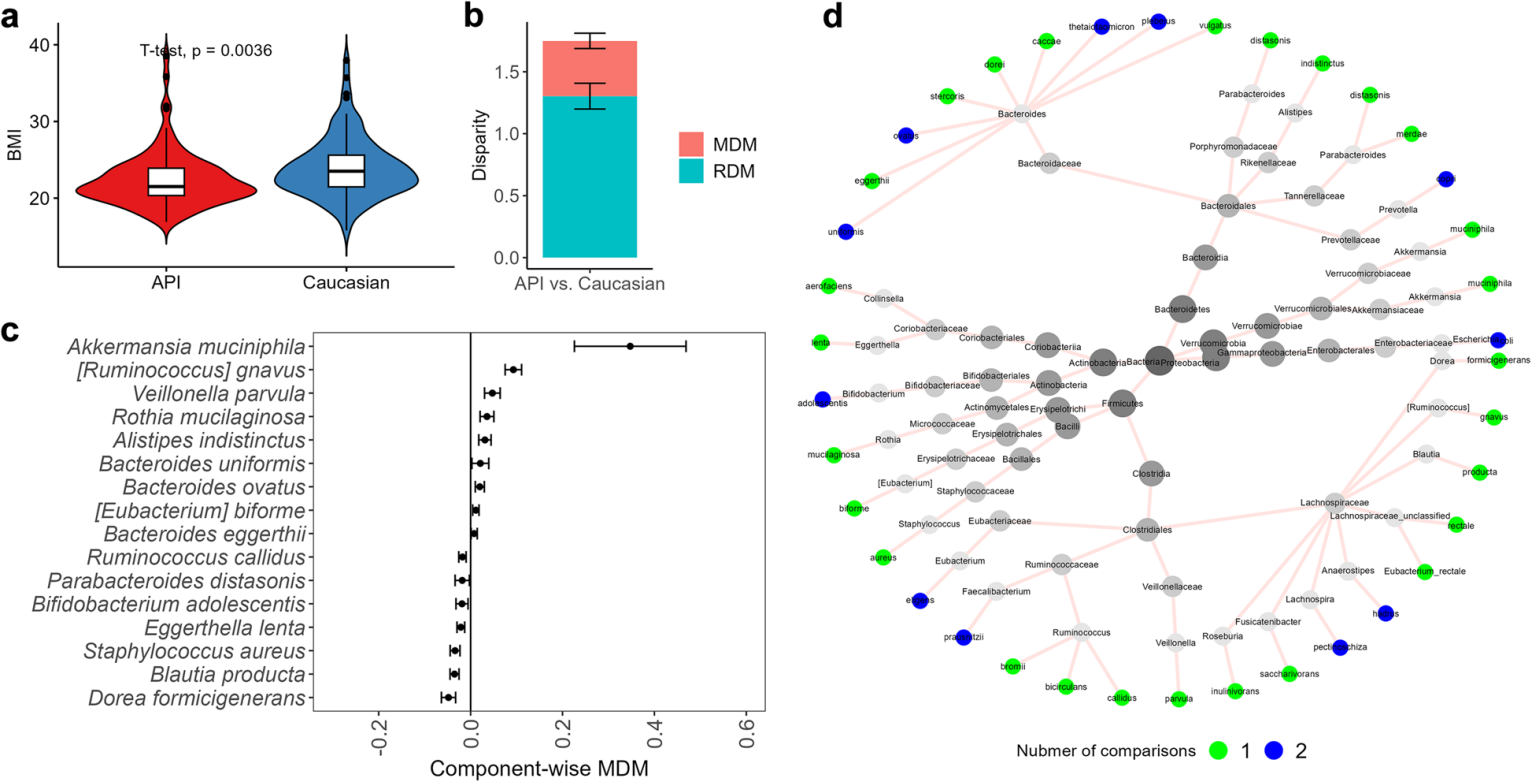
Health disparity analyses in the matched APIs and Caucasians from the AGP dataset. **a** Violin plots of BMI in the matched APIs and Caucasians from the AGP dataset. **b** The average and standard error of MDM and RDM of BMI in the API- Caucasian comparison based on the splitting strategy with 50 times of repetitions. **c** Component-wise point and 95% CI estimates of ${MDM}_{j}$ for the identified species that have mediation effects on the differences of BMI between matched APIs and Caucasians from the AGP dataset. 95% CI estimates of ${MDM}_{j}$ were calculated based on the splitting strategy with 50 times of repetitions. **d** The taxonomic relationship of the species playing mediation effects in the disparity of BMI among China-USA, China-UK, and API-Caucasian comparisons. The tree figure was generated by Metacoder [55]. From the outer to the center, taxonomic ranks are species, genus, family, order, class, phylum, and kingdom (Bacteria), respectively. For each species, color represents the number of comparisons that identify it among China-USA, China-UK, and API-Caucasian comparisons. APIs Asian or Pacific Islanders

Significant CMD testing results show that there is at least one species playing a mediating role in the disparity of BMI between Chinese and USA subjects and Chinese and UK subjects. Figure 3b reports 15 species and 18 species further identified by SparseMCMM_HD, with the point and 95% CI estimates for their mediation effects on the regional differences of BMI between China and USA and between China and UK, respectively. Among the twelve overlapping species identified in both matched datasets (Fig. 3b and c), five species—*Anaerostipes hadrus*, *Bacteroides plebeius*, *Bacteroides thetaiotaomicron*, *Escherichia* coli, and *Eubacterium rectale*—play consistent positive mediating roles in regional disparity in BMI for Chinese compared to USA subjects and for Chinese compared to UK subjects. The relative evaluation of these five species in terms of their relative abundances (Fig. 4a) and their associations with BMI (Fig. 4b) are quite similar between two independent studies: China-USA comparison and China-UK comparison, which validates their mediating roles in the regional disparity on BMI. Confirming with the published studies, *B. plebeius* and *B. thetaiotaomicron* belong to the same genus *Bacteroides*, and all play important roles in human metabolism and have been linked with diet-induced obesity, by improving whole-body glucose disposal, promoting lipid digestion and absorption, and degrading host-derived carbohydrates [38–41]. *B. thetaiotaomicron* also possesses glycine lipid biosynthesis pathway (Figure S4). *A. hadrus*, *E. coli*, and *E. rectale* also have been reported by multiple studies that they contribute to or are associated with the BMI or obesity [42–44]. On the other hand, four species play mediating roles in BMI but with the opposite directions between China-USA comparison and China-UK comparison that reflects the distinguishing characteristics between USA and UK (Figure S5). This is not surprising considering the microbial profile is inherently dynamic and ethnically or geographically specific. Moreover, there are six and nine unique species identified in the China-USA and China-UK comparisons, respectively (Figures S6 and S7). Most of these study-specific species have been reported being associated with BMI, obesity, or metabolic disorders [44–53]. Notably, *Anaerostipes hadrus*, *Fusicatenibacter saccharivorans*, *Lachnospira pectinoschiza*, and *Roseburia inulinivorans* belong to family *Lachnospiraceae* (Fig. 5d), which is related to metabolic syndrome and obesity and whose controversial role has been discussed across different studies [54].

### Results for AGP

#### Matched dataset

After performing PSM, as described in Section S2, 98 Caucasians and 98 APIs are matched. Figures S8 and S9 show that the matched Caucasians and APIs have very similar propensity scores (SMD = 0.005 for the matched subjects vs. SMD = 1.033 for the raw subjects), indicating that the confounding effects are well controlled. With this well-matched dataset, Fig. 5a shows that the Caucasian group has a significantly higher BMI (23.96 [3.92]), compared to the API group (22.38 [3.59]), as observed in the other studies [56, 57].

#### Community level results

Caucasians and APIs have distinct microbial profiles in terms of community diversity. For alpha diversity, Caucasians have higher microbial richness and evenness as measured by Observed, Shannon, and Simpson diversities (*p* value = $3.1 \times {10}^{-5}$, $1.5 \times {10}^{-4}$, and $3.9 \times {10}^{-3}$, respectively, Figure S10a). For Beta diversity, Bray–Curtis dissimilarity and Jensen-Shannon divergence both show that Caucasian samples have different community structures compared to API samples (PERMANOVA *p* value = 0.0036 and 0.0012, respectively, Figure S10b).

#### Taxon-level analysis

The above community level results indicate that the microbiome may play a mediating role in the ethnic diversity of BMI. To investigate this assumption, we perform the proposed SparseMCMM_HD on this matched dataset. With the filtering criteria described in Section S4, 28 species are included in the following taxon-level analysis.

We found that the average ODM of BMI between Caucasians and APIs is 1.75 (Fig. 5b). Microbiome plays a significant role in mediating the ethnic disparity of BMI indicated by the test results of both OMD (*p* value = 0.035) and CMD (*p* value = 0.036). The average manipulable disparity measure MDM is 0.45. This suggests that the difference of microbiome profiles contributes to 25.71% of ODM, which would be eliminated if the microbiome profiles between the Caucasians and APIs were identical.

We further identified 16 species playing mediating roles in the ethnic disparity of BMI between the Caucasians and APIs (Fig. 5c). Nine species (*[Ruminococcus] gnavus*, *Rothia mucilaginosa*, *Bacteroides uniformis*, *Bacteroides eggerthii*, *Bacteroides ovatus*, *Veillonella parvula*, *[Eubacterium] biforme*, *Akkermansia muciniphila*, *Alistipes indistinctus*) mediate positively on the ethnic disparity of BMI, meanwhile, seven species (*Dorea formicigenerans*, *Staphylococcus aureus*, *Blautia product*, *Bifidobacterium adolescentis*, *Parabacteroides distasonis*, *Eggerthella lenta*, *Ruminococcus callidus*) play negative mediating roles. Remarkably, there are six common species *A. muciniphila*, *B. ovatus*, *B. uniformis*, *B. adolescentis*, *F. prausnitzii*, and *P. distasonis* identified by China-USA or China-UK comparison illustrated in the previous subsection (Fig. 5d). Literature reveals that all identified species are associated with the BMI or obesity [44–52].

Collectively, the findings in the matched China vs. USA, China vs. UK, and API vs. Caucasian datasets show that the microbiome is an important mediator in the regional or ethnic disparity of BMI and they substantially shed light on how to reduce the disparity of BMI. The identified microbial agents can be used as the potential therapeutic target for the treatment based on microbiota modulation in the future.

## Discussion

The emerging evidence highlights the potential of microbiome in understanding health disparity. In this paper, we proposed a mediation analytical framework, SparseMCMM_HD, to investigate the microbiome’s role in health disparity. Considering a health disparity framework with three components: a non-manipulable exposure (e.g., ethnicity or region), the microbiome as mediator, and a continuous outcome, the proposed SparseMCMM_HD deciphers the overall health disparity of the non-manipulable exposure on the outcome into two components: MDM that would be eliminated by equalizing microbiome profiles and RDM that would remain and could not be explained through the microbiome. Remarkably, MDM paves a viable path towards reducing health disparity with microbial modulation. Similar to the illustration in SparseMCMM, SparseMCMM_HD identified the signature causal microbes and examined whether the overall or component-wise MDM is significantly non-zero, respectively. Moreover, we elucidated the relevance and novelties of SparseMCMM_HD in comparison to SparseMCMM in Section S5.

Due to the identifiability assumptions of the causal interpretation of microbial contributions to health disparities, it is vital to control confounding effects. In three BMI applications, we employed PSM to remove the confounding effects by selecting matched subsets in which the distribution of confounders was notably comparable and then performed the proposed SparseMCMM_HD framework. The utilization of SparseMCMM_HD in two matched datasets, the curatedMetagenomicData 3.4.2 package and the AGP dataset, depicts an explicit causal path among region or ethnicity, microbiome, and BMI. These findings confirm not only that the microbiome is differentially distributed across ethnicities or regions and affects the BMI, but also that the differential microbiome profile contributes to the disparities in BMI across ethnicities or regions. The identified microbial signatures potentially aid in the development of personalized medication or nutrition for the reduction of obesity disparity by targeting the microbial profiles.

It is not surprising that the proportion of disparities in BMI explained by the microbiome profiles is not large (20 ~ 30%) in all three applications, due to the heritable and polygenic nature of BMI [58, 59]. Further investigations to integrate the microbiome profile and genetic factors are necessary to better understand disparity in BMI. However, we here emphasize that the proposed SparseMCMM_HD is a rigorous and validated causal mediation framework and has preeminent potential to identify the microbiome’s roles in much broader health disparity studies.

Recently, several other microbial mediation methods have been proposed, such as CMM [60], MedTest [61], Zhang et al. [62], LDM-med [63], and MarZIC [64], in a typical three-factor (a manipulable exposure, microbiome as mediator, and outcome) study design. Considering distinct model assumptions and characteristics, a few recent benchmark studies [12, 60–64] show that there is no method performing consistently and accurately better than others in all circumstances. However, since the assumptions for model identification in health disparity are weaker than those for the causal mediation effects in the manipulable exposure-mediator-outcome framework [24], it is expected that the idea of how the proposed SparseMCMM_HD framework rigorously defines, quantifies, and tests health disparity measures as an extension of SparseMCMM [12] can provide insight into extending these available mediation models to investigate the microbiome’s role in health disparity. Then, a useful path forward will be to mutually employ these multiple and complementary methods to better characterize the microbiome’s role in health disparity by capitalizing their distinct assumptions and strengths.

Our study has several limitations. First, similar to discussions in SparseMCMM [12], SparseMCMM_HD takes microbiome data at a fixed time point into the proposed frame and is limited to accommodate the dynamic nature of microbiome. Second, the proposed SparseMCMM_HD currently deals with disparity in a continuous outcome. Given the fact that multiple binary or categorical outcomes are disproportionately prevalent across ethnicities or regions [65–67], it will be worthwhile to extend the current framework to handle categorical outcomes. Third, microbiome studies typically characterize both taxonomic and functional profiles of the microbes within a community. Functional profile is generally thought to be more closely linked with human health and disease. Identifying the functional profile in the health disparity is of high practical value [68]. Fourth, the application of a splitting strategy is constrained due to the inference-prediction tradeoff, particularly when dealing with smaller sample sizes. Fifth, due to the limited metadata available in our three comparison datasets, the application of PSM may not adequately account for latent variables that exert confounding effects on the health disparities analysis. Future research is needed for further validation and comprehensive clinical assessments.

## Conclusions

This paper elucidates the role of microbiome in health disparity by providing a causal mediation analytic framework for investigating the relationship among ethnicity or region, microbiome, and the outcome of interest under the counterfactual framework. The proposed SparseMCMM_HD framework is a useful tool to investigate the underlying biological mechanism of health disparity and disentangles the substantial contributions of microbiome to health disparity. The applications of SparseMCMM_HD in the disparity of BMI across ethnicities and regions uncover the microbial mediating roles in reducing the disparities of BMI and improving health equality.

## Supplementary Information

**Additional file 1: Section S1.** Derivations for MDM and RDM expressions. **Section S2.** Propensity score matching (PSM). **Section S3.** Metadata curation in the AGP. **Section S4.** Taxon-level alignment. **Section S5.** SparseMCMM_HD’s distinctions and novelties in comparison to SparseMCMM. **Section S6.** SparseMCMM_HD web app instruction. **Table S1.** Comparisons between SparseMCMM_HD and SparseMCMM (the blue shaded sections indicate the difference). **Figure S11.** The Workflow panel in the SparseMCMM_HD web app. **Figure S12**. The left panel of the SparseMCMM_HD web app. **Figure S13.** PSM results displayed in the PSM panel in China-USA comparison analysis. **Figure S14.** Group comparison results displayed in the Association analysis panel in the matched China-USA dataset in China-USA comparison analysis. **Figure S15.** Results displayed in the Health disparity analysis panel in the matched China-USA dataset in China-USA comparison analysis.

**Additional file 2: Figure S1.** Flowcharts for data pre-processing in the AGP dataset. a Pre-processing for all covariates. b The sample breakdown for the disparity analysis. **Figure S2.** Plots of standardized mean differences before and after propensity score matching for the datasets from the curatedMetagenomicData package [28]. a Comparison between Chinese and USA subjects. b Comparison between Chinese and UK subjects. **Figure S3.** Histogram plots of propensity score before and after propensity score matching for the datasets from the curatedMetagenomicData package [28]. a Comparison between Chinese and USA subjects. b Comparison between Chinese and UK subjects. **Figure S4.** Glycine lipid biosynthesis pathway generated based on MetaCyc database (https://metacyc.org/). The gene from B.thetaiotaomicro is located in an operon together with a second gene, glsA, which encodes the second enzyme of the pathway, an O-acyltransferase that forms the diacylated compound. **Figure S5.** The species with opposite mediation directions in the disparity of BMI between China-USA and China-UK comparisons. a Violin plots illustrating the relative abundances of these identified species in the matched Chinese and USA samples, and the matched Chinese and UK samples, respectively. b Scatterplots of BMI and the relative abundances of these identified species in the matched Chinese and USA samples, and the matched Chinese and UK samples, respectively. **Figure S6.** The species playing mediation roles in the disparity of BMI in the comparison between Chinese and USA subjects only. a Violin plots illustrating the relative abundances of these identified species in the matched Chinese and USA samples. b Scatterplots of BMI and the relative abundances of these identified species in the matched Chinese and USA samples. **Figure S7.** The species playing mediating roles in the disparity of BMI in the comparison between Chinese and UK subjects only. a Violin plots illustrating the relative abundances of these identified species in the matched Chinese and UK samples. b Scatterplots of BMI and the relative abundances of these identified species in the matched Chinese and UK samples. **Figure S8.** Plots of standardized mean differences before and after propensity score matching for the comparison between the API and Caucasian samples from the AGP dataset. API: Asian or Pacific Islander. **Figure S9.** Histogram plots of propensity score before and after propensity score matching for the comparison between the API and Caucasian samples from the AGP dataset. API: Asian or Pacific Islander. **Figure S10.** Association analyses in the AGP dataset. a Violin plots of alpha diversities including Observed, Shannon, and Simpson indices in the matched API and Caucasian samples. b PCoA plots using Bray–Curtis dissimilarity and Jensen–Shannon divergence in the matched API and Caucasian samples. API: Asian or Pacific Islander.

## Data Availability

All relevant datasets are publicly available. The data used in investigations of the regional difference of BMI in the China group compared to the United States (USA) and United Kingdom (UK) groups can be downloaded from the curatedMetagenomicData 3.4.2 package [28]. The data used in investigations of the ethnic difference in BMI between Caucasians and Asian or Pacific Islanders are from the American Gut Project. Their raw data and metadata are publicly available on the FTP website (ftp://ftp.microbio.me/AmericanGut/). Version 07/29/2016 is used in our analyses. SparseMCMM R package is available at https://github.com/chanw0/SparseMCMM. The interactive web app for the proposed SparseMCMM_HD framework is available at https://chanw0.shinyapps.io/sparsemcmm_hd/, and its detailed instructions are shown in Section S6.

## Abbreviations

AGP: American gut project
API: Asian or Pacific Islander
BMI: Body mass index
MDM: Manipulable disparity measure
ODM: Overall disparity measure
PERMANOVA: Permutational multivariate analysis of variance
PSM: Propensity score matching
RDM: Residual disparity measure
SparseMCMM: Sparse microbial causal mediation model
SparseMCMM_HD: SparseMCMM for health disparity
SMD: Standardized mean difference
UK: United Kingdom
USA: United States
