# Supplementary material for: A microbial causal mediation analytic tool for health disparity and applications in body mass index
Source: Microbiome. 2023 Jul 27;11:164. doi: 10.1186/s40168-023-01608-9 (PMC10373330; doi:10.1186/s40168-023-01608-9)
Supplement: Supplementary file 2 — Additional file 1: Section S1. Derivations for MDM and RDM expressions. Section S2. Propensity score matching (PSM). Section S3. Metadata curation in the AGP. Section S4. Taxon-level alignment. Section S5. SparseMCMM_HD’s distinctions and novelties in comparison to SparseMCMM. Section S6. SparseMCMM_HD web app instruction. Table S1. Comparisons between SparseMCMM_HD and SparseMCMM (the blue shaded sections indicate the difference). Figure S11. The Workflow panel in the SparseMCMM_HD web app. Figure S12. The left panel of the SparseMCMM_HD web app. Figure S13. PSM results displayed in the PSM panel in China-USA comparison analysis. Figure S14. Group comparison results displayed in the Association analysis panel in the matched China-USA dataset in China-USA comparison analysis. Figure S15. Results displayed in the Health disparity analysis panel in the matched China-USA dataset in China-USA comparison analysis. [file 40168_2023_1608_MOESM1_ESM.docx]

# **Section S1 Derivations for MDM and RDM expressions**

With these sufficient identifiability assumptions [1, 2] and the models (1)-(2) proposed in the SparseMCMM_HD framework in the main text, the manipulable disparity measure MDM is defined as

$$\begin{matrix} \mathrm{MDM} & =E\left[ E[Y_{\boldsymbol{M}_{\boldsymbol{x}}(1)}|R=1,\boldsymbol{x}] \right]-E\left[ E[Y_{\boldsymbol{M}_{\boldsymbol{x}}(0)}|R=1,\boldsymbol{x}] \right] \\ & =\int E[Y_{\boldsymbol{M}_{\boldsymbol{x}}(1)}|R=1,\boldsymbol{M}_{\boldsymbol{x}}(1),\boldsymbol{x}]dF(\boldsymbol{M}_{\boldsymbol{x}}(1))-\int E[Y_{\boldsymbol{M}_{\boldsymbol{x}}(0)}|R=1,\boldsymbol{M}_{\boldsymbol{x}}(0),\boldsymbol{x}]dF(\boldsymbol{M}_{\boldsymbol{x}}(0)) \\ & =\int E[Y|R=1,\boldsymbol{M},\boldsymbol{x}]dF(\boldsymbol{M}|R=1,\boldsymbol{x})-\int E[Y|R=1,\boldsymbol{M},\boldsymbol{x}]dF(\boldsymbol{M}|R=0,\boldsymbol{x}) \\ & =\int\left\{ \alpha_{0}+\alpha_{R}+\boldsymbol{\alpha}_{X}^{T}\boldsymbol{x}+\boldsymbol{\alpha}_{M}^{T}[log(\boldsymbol{M})]+\boldsymbol{\alpha}_{C}^{T}log(\boldsymbol{M}) \right\}dF(\boldsymbol{M}|R=1,\boldsymbol{x})- \\ & \int\left\{ \alpha_{0}+\alpha_{R}+\boldsymbol{\alpha}_{X}^{T}\boldsymbol{x}+\boldsymbol{\alpha}_{M}^{T}[log(\boldsymbol{M})]+\boldsymbol{\alpha}_{C}^{T}log(\boldsymbol{M}) \right\}dF(\boldsymbol{M}|R=0,\boldsymbol{x}) \\ & =(\boldsymbol{\alpha}_{M}^{T}+\boldsymbol{\alpha}_{C}^{T})\{E[log(\boldsymbol{M})|R=1,\boldsymbol{x}]-E[log(\boldsymbol{M})|R=0,\boldsymbol{x}]\} \\ & =\sum_{j=1}^{J} (\alpha_{Mj}+\alpha_{Cj})\left\{ E\left[ \log\left( M_{j} \right) | R=1,\boldsymbol{x} \right]-E\left[ \log\left( M_{j} \right) | R=0,\boldsymbol{x} \right] \right\}. \end{matrix}$$

The residual disparity measure RDM is defined as

$$\begin{matrix} \mathrm{RDM} & =E\left[ E\left[ Y_{\mathbf{M}_{\boldsymbol{x}}\left( 0 \right)} | R=1,\boldsymbol{x} \right]-E\left[ Y_{\mathbf{M}_{\boldsymbol{x}}\left( 0 \right)} | R=0,\boldsymbol{x} \right] \right] \\ & =\int\left\{ E\left[ Y_{\mathbf{M}_{\boldsymbol{x}}\left( 0 \right)} | R=1,\boldsymbol{M}_{\boldsymbol{x}}\left( 0 \right),\boldsymbol{x} \right]-E\left[ Y_{\boldsymbol{M}_{\boldsymbol{x}}\left( 0 \right)} | R=0,\boldsymbol{M}_{\boldsymbol{x}}\left( 0 \right),\boldsymbol{x} \right] \right\}dF\left( \boldsymbol{M}_{\boldsymbol{x}}\left( 0 \right) \right) \\ & =\int\left\{ E\left[ Y | R=1,\boldsymbol{M},\boldsymbol{x} \right]-E\left[ Y | R=0,\boldsymbol{M},\boldsymbol{x} \right] \right\}dF\left( \boldsymbol{M} | R=0,\boldsymbol{x} \right) \\ & =\int\{\alpha_{0}+\alpha_{R}+\boldsymbol{\alpha}_{X}^{T}\boldsymbol{x}+\boldsymbol{\alpha}_{M}^{T}[\text{log}(\boldsymbol{M})]+\boldsymbol{\alpha}_{C}^{T}\text{log}(\boldsymbol{M})-\alpha_{0}-\boldsymbol{\alpha}_{X}^{T}\boldsymbol{x}- \\ & \boldsymbol{\alpha}_{M}^{T}[\text{log}(\boldsymbol{M})]\}dF(\boldsymbol{M}|R=0,\boldsymbol{x}) \\ & =\int\left\{ \alpha_{R}+\boldsymbol{\alpha}_{C}^{T}\left[ \text{log}\left( \boldsymbol{M} \right) \right] \right\}dF\left( \boldsymbol{M} | R=0,\boldsymbol{x} \right) \\ & =\alpha_{R}+\boldsymbol{\alpha}_{C}^{T}E\left[ \text{log}\left( \boldsymbol{M} \right) | R=0,\boldsymbol{x} \right] \\ & = \alpha_{R}+\boldsymbol{\alpha}_{C}^{T}E\left[ \text{log}\left( \boldsymbol{M} \right) | R=0,\boldsymbol{x} \right]=\alpha_{R}+\sum_{j=1}^{J} \alpha_{Cj}E\left[ \log\left( M_{j} \right) | R=0,\boldsymbol{x} \right]. \end{matrix}$$

Therefore the overall disparity measure is

$$\begin{matrix} \mathrm{ODM} & =MDM+RDM \\ & =\alpha_{R}+\sum_{j=1}^{J} (\alpha_{Mj}+\alpha_{Cj})E\left[ \log\left( M_{j} \right) | R=1,\boldsymbol{x} \right]-\sum_{j=1}^{J} \alpha_{Mj}E\left[ \log\left( M_{j} \right) | R=0,\boldsymbol{x} \right], \end{matrix}$$

where $E\left[ \log\left( M_{j} \right) | R=r,\boldsymbol{x} \right]=\psi\left[ \gamma_{j}\left( R=r,\boldsymbol{x} \right) \right]-\psi\left[ \sum_{m=1}^{J} \gamma_{m}\left( R=r,\boldsymbol{x} \right) \right]$, $\gamma_{j}\left( R=r,\boldsymbol{x} \right)=\exp\left( \beta_{0j}+\beta_{Rj}r+\boldsymbol{\beta}_{Xj}^{T}\boldsymbol{x} \right)$, $r=0$ or 1, and $\psi\left( \cdot\right)=\frac{d}{dx}\ln\left( \Gamma\left( x \right) \right)$ is the digamma function, with given covariates $\boldsymbol{x}\mathbf{.}$

# **Section S2 Propensity score matching (PSM)**

As discussed in the Methods section, it is necessary to control confounding covariates before performing the proposed method SparseMCMM_HD to investigate the relationship among a non-manipulable exposure, microbiome, and the outcome in the health disparity research. In our BMI applications using studies from curatedMetagenomicData and AGP, several covariates are associated with the microbiome profile or BMI [3-5]. PSM [6], which is a commonly used method in biomedical research to create a balanced covariate distribution between two groups, is employed to remove the bias of confounders from influencing the relationship among race or region, microbiome, and BMI. Following suggestions in [7], we adopt nearest neighbor matching within a specified caliper distance, where the matched scale is the logit of the propensity score, and the caliper distance is 0.2 × the standard deviation of the matched scale, and the matching ratio is 1:1 without replacement. The matched data will then be used to quantify RDM, MDM, and ODM, and examine whether the microbiome could reduce the health disparity between two non-manipulable exposure groups. The control for confounding covariates procedure has been included as a preprocessing step in the proposed SparseMCMM_HD analytic pipeline (<https://chanw0.shinyapps.io/sparsemcmm_hd/>).

# **Section S3 Metadata curation in the AGP**

There are more than 400 self-reported covariates in the AGP. Principally, pre-processing procedures are implemented to prepare the self-reported covariates ready for the subsequent analysis, such as missing value identification, duplicated or highly correlated variables exclusion, variable collapse, and quality control. Specifically, value Unknown, no_data, Not sure, or blank is regarded as a missing value (NA). When a variable has multiple measurements, a continuous measurement is preferred. For example, age has both continuous and categorical measurements, and a continuous measure is selected. False is the same as No, as well as True is the same as Yes. For the ordinal variables with more than two categories, if the category with a less than 10% proportion is collapsed into its neighborhood category. For example, exercise frequency has five categories: Never (1.95%), Rarely (a few times/month; 9.59%), Occasionally (1-2 times/week; 21.51%), Regularly (3-5 times/week; 42.61%), and Daily (24.25%). After collapsing, exercise frequency has four categories: Never or Rarely (11.54%), Occasionally (21.51%), Regularly (42.61%), and Daily (24.25%). Smoking frequency also has five categories: Never (95.18%), Rarely (2.37%), Occasionally (0.75%), Regularly (0.46%), and Daily (0.91%), and is combined into two categories: Never (95.18%) and Smoking (4.49%). In terms of quality control, covariates are further excluded from the analysis, if their missing proportions are larger than 90% in the population or larger than 20% in the Asian Pacific Islander group, or if one category is dominant in the population with a larger than 90% proportion for categorical variables. Then these pre-curated covariates are used in the analytical step regarding whether they potentially are confounding.

# **Section S4 Taxon-level alignment**

In this paper, we perform the proposed SparseMCMM_HD at the species rank to investigate whether the species play a mediating role in the region or racial difference of BMI overall and individually. For the shotgun metagenomics samples in the curatedMetagenomicData and the 16S rRNA samples in the AGP, species aligned to the bacterial kingdom are considered. Following phyloseq’s common data processing criteria [8], species are further removed if they are not present more than 3 times in at least 20% of the samples, or their average relative abundances are less than 1%.

**Section S5 SparseMCMM_HD’s distinctions and novelties in comparison to SparseMCMM**

Although the final mathematical expressions of manipulable disparity measure (MDM) and residual disparity measure (RDM) defined in the SparseMCMM_HD are the same as formulas of causal direct effect (DE) of treatment and mediation effect (ME) through microbiome correspondingly on the outcome, developed in our SparseMCMM [9], the proposed SparseMCMM_HD framework is intrinsically different from SparseMCMM, and have several novel contributions. Table S1 summarizes the comparisons between SparseMCMM_HD and SparseMCMM in mediation framework, disparity or mediation definition, and model assumption and derivation. Mainly, first, they address two different research questions arising from two different study designs. SparseMCMM_HD aims to explore the extent of health disparities in the outcome that could be reduced if the gut microbial profile was modified, in a three-factor (a non-manipulable exposure i.e., ethnicity or region, microbiome as mediator, and outcome) observational study design, while SparseMCMM aims to identify the treatment effect on the outcome through microbiome in a three-factor (a randomized exposure/treatment, microbiome as mediator, and outcome) randomization study design. Second, the factors used to interpret the causality are different (highlighted in red in the framework illustration). As VanderWeele and Robinson discussed in [1] and we discuss in the main text, SparseMCMM_HD interprets the causality of health inequality by the hypothesized intervention effect on the manipulable microbiome mediators, while SparseMCMM interprets causal direct and mediation effects by the hypothesized intervention on the exposure/treatment. Third, starting with different causal interpretations, the definitions of MDM and RDM are different from ME and DE, respectively. MDM is defined as the portion of disparity that would be eliminated by equalizing microbiome profiles between comparison and reference groups, and RDM is defined as the portion that would remain even after microbiome profiles between comparison and reference groups were equalized. While, ME is the expected change in outcome if the exposure were controlled at the comparison group (T=1), but the mediators were changed from the value they would have taken had the exposure been the reference group (T=0) to the value they would have taken had the exposure been the comparison group (T=1). DE is the expected difference between the counterfactual outcome that would have taken had the exposure been set to the comparison group (T=1) and the counterfactual outcome that would have taken had the exposure been set to the reference group (T=0), while the mediators were set to the values they would have taken had the exposure been set to the reference group (T=0). Fourth, with the counterfactual notations, MDM and RDM have different mathematical formula compared with ME and DE. In SparseMCMM_HD, the microbiome mediators are random variables and the counterfactual outcome constructed in MDM and RDM is determined by these mediators. In SparseMCMM, the treatment is a random variable and the counterfactual outcome constructed in ME and DE is determined by it.

Due to microbiome’s unique data structures, SparseMCMM_HD employs the same causal mediation model (linear log-contrast regression and Dirichlet regression) used in SparseMCMM [9], to describe the relationship among non-manipulable exposure, microbiome, and outcome. However, SparseMCMM_HD needs different assumptions to identify and estimate MDM and RDM under the counterfactoral framework, compared with assumptions for identification and estimation of DE and ME defined in SparseMCMM [9]. Given the above causal mediation model and sufficient assumptions, through mathematical deviations, the estimated expressions of MDM and RDM are just the same as formulas of DE and ME.

Despite the final expressions of MDM and RDM and DE and ME are correspondingly the same, the proposed SparseMCMM_HD framework has sound novelties regarding causal interpretation, rigorous conceptualizations, and quantifications of health disparity measures (MDM and RDM) through microbiome under the counterfactual framework.

As discussed in Discussion section, several other microbial mediation methods have been proposed in a typical three-factor (a manipulable exposure, microbiome as mediator, and outcome) study design, such as CMM [10], MedTest [11], Zhang, et al. [12], LDM-med [13], and MarZIC [14]. The novelties of SparseMCMM_HD provide insight into extending these available mediation models to investigate microbiome’s role in health disparities, despite distinct model assumptions and characteristics. Thus, a useful path forward will be to mutually employ these multiple and complimentary methods to better characterize the microbiome’s role in health disparities by capitalizing their distinct assumptions and strengths.

Table S1. Comparisons between SparseMCMM_HD and SparseMCMM (the blue shaded sections indicate the difference).

|  |  | **SparseMCMM_HD** | **SparseMCMM** |
| --- | --- | --- | --- |
| Mediation framework | Study design | A three-factor (a non-manipulable exposure, microbiome as mediator, and outcome) observational study design | A three-factor (a randomized exposure/ treatment, microbiome as mediator, and outcome) randomization study design |
|  | Framework illustration | 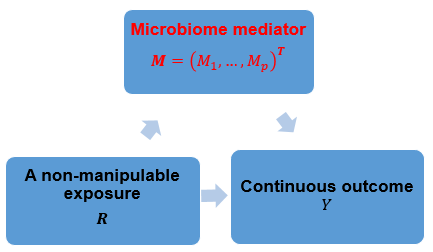 | 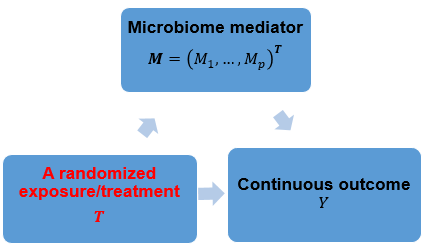 |
| Disparity or mediation definition | Factor hypothetically being intervened | Microbiome profile | Exposure/treatment |
|  | Definition under the counterfactual framework | The overall disparity measure (ODM) on the outcome is decomposed into manipulable disparity measure (MDM) and residual disparity measure (RDM).   - MDM represents the portion of disparity that would be eliminated by equalizing microbiome profiles between comparison and reference groups; - RDM represents the portion that would remain even after microbiome profiles between comparison and reference groups were equalized. | The total effect of the treatment on the outcome (TE) is the summation of mediation effect (ME) and direct effect (DE).   - ME is the expected change in outcome if the exposure were controlled at the comparison group (T=1), but the mediators were changed from the value they would have taken had the exposure been the reference group (T=0) to the value they would have taken had the exposure been the comparison group (T=1). - DE is the expected difference between the counterfactual outcome that would have taken had the exposure been set to the comparison group (T=1) and the counterfactual outcome that would have taken had the exposure been set to the reference group (T=0), while the mediators were set to the values they would have taken had the exposure been set to the reference group (T=0). |
|  | Math definition | $\mathrm{MDM}=E\left[ E[Y_{\boldsymbol{M}_{\boldsymbol{x}}(1)}\vert R=1,\boldsymbol{x}] \right]-E\left[ E[Y_{\boldsymbol{M}_{\boldsymbol{x}}(0)}\vert R=1,\boldsymbol{x}] \right]$  $\mathrm{RDM}=E\left[ E[Y_{\boldsymbol{M}_{\boldsymbol{x}}(0)}\vert R=1,\boldsymbol{x}]-E[Y_{\boldsymbol{M}_{\boldsymbol{x}}(0)}\vert R=0,\boldsymbol{x}] \right]$ | $\mathrm{ME}=E\left[ Y_{{T=1, \boldsymbol{M}}_{\boldsymbol{x}}\left( T=1 \right)}-Y_{{T=1, \boldsymbol{M}}_{\boldsymbol{x}}\left( T=0 \right)}\vert\boldsymbol{x} \right]$  $\mathrm{DE}=E\left[ Y_{{T=1, \boldsymbol{M}}_{\boldsymbol{x}}\left( T=0 \right)}-Y_{{T=0, \boldsymbol{M}}_{\boldsymbol{x}}\left( T=0 \right)}\vert\boldsymbol{x} \right]$ |
| Model assumption and derivation | Causal mediation model | Linear log-contrast regression and Dirichlet regression | Linear log-contrast regression and Dirichlet regression |
|  | Identifiability assumptions | 1) The effect of non-manipulable exposure *R* on outcome *Y* is unconfounded conditional on all covariates $\boldsymbol{X}$, i.e., $Y\coprod R\boldsymbol{\vert X}$;  2) The effects of mediator ***M*** on outcome *Y* are unconfounded conditional on the non-manipulable exposure *R* and all covariates $\boldsymbol{X}$, i.e., $Y\coprod\boldsymbol{M}\boldsymbol{\vert}R,\boldsymbol{X}$. | 1) The effect of treatment *T* on outcome *Y* is unconfounded conditional on all covariates $\boldsymbol{X}$, i.e., $Y\coprod T\boldsymbol{\vert X}$;  2) The effects of mediator ***M*** on outcome *Y* are unconfounded conditional on treatment *T* and all covariates $\boldsymbol{X}$, i.e., $Y\coprod\boldsymbol{M}\boldsymbol{\vert}T,\boldsymbol{X}$.  3) The effect of treatment *T* on mediator ***M*** is unconfounded conditional on all covariates $\boldsymbol{X}$, i.e., $\boldsymbol{M}\coprod T\boldsymbol{\vert X}$;  4) There is no unmeasured confounders for the relationship between mediator and outcome that can be affected by treatment, i.e., $Y(t, \boldsymbol{m})\coprod\boldsymbol{M}\boldsymbol{(}t^{*}\boldsymbol{)\vert X}$ for all levels of $t$, $t^{*}$, and $\boldsymbol{m}$. |
|  | Derivation* | $\mathrm{MDM}=E\left[ E\left[ Y_{\boldsymbol{M}_{\boldsymbol{x}}\left( 1 \right)} \vert R=1,\boldsymbol{x} \right] \right]-E\left[ E\left[ Y_{\boldsymbol{M}_{\boldsymbol{x}}\left( 0 \right)} \vert R=1,\boldsymbol{x} \right] \right]$  =$\int E\left[ Y_{\boldsymbol{M}_{\boldsymbol{x}}\left( 1 \right)} \vert R=1,\boldsymbol{M}_{\boldsymbol{x}}\left( 1 \right),\boldsymbol{x} \right]dF\left( \boldsymbol{M}_{\boldsymbol{x}}\left( 1 \right) \right)$  $-\int E[Y_{\boldsymbol{M}_{\boldsymbol{x}}(0)}\vert R=1,\boldsymbol{M}_{\boldsymbol{x}}(0),\boldsymbol{x}]dF(\boldsymbol{M}_{\boldsymbol{x}}(0))$  =$\int E[Y\vert R=1,\boldsymbol{M},\boldsymbol{x}]dF(\boldsymbol{M}\vert R=1,\boldsymbol{x})$  $-\int E[Y\vert R=1,\boldsymbol{M},\boldsymbol{x}]dF(\boldsymbol{M}\vert R=0,\boldsymbol{x})$  $RDM=E\left[ E\left[ Y_{\boldsymbol{M}_{\boldsymbol{x}}\left( 0 \right)} \vert R=1,\boldsymbol{x} \right]-E\left[ Y_{\boldsymbol{M}_{\boldsymbol{x}}\left( 0 \right)} \vert R=0,\boldsymbol{x} \right] \right]$  =$\int\left\{ E\left[ Y_{\boldsymbol{M}_{\boldsymbol{x}}\left( 0 \right)} \vert R=1,\boldsymbol{M}_{\boldsymbol{x}}\left( 0 \right),\boldsymbol{x} \right]- E\left[ Y_{\boldsymbol{M}_{\boldsymbol{x}}\left( 0 \right)} \vert R=0,\boldsymbol{M}_{\boldsymbol{x}}\left( 0 \right),\boldsymbol{x} \right] \right\}dF\left( \boldsymbol{M}_{\boldsymbol{x}}\left( 0 \right) \right)$  $=\int\left\{ E\left[ Y \vert R=1,\boldsymbol{M},\boldsymbol{x} \right]- E\left[ Y \vert R=0,\boldsymbol{M},\boldsymbol{x} \right] \right\}dF\left( \boldsymbol{M} \vert R=0,\boldsymbol{x} \right)$ | $\mathrm{ME}=E\left[ Y_{{T=1, \boldsymbol{M}}_{\boldsymbol{x}}\left( T=1 \right)}-Y_{{T=1, \boldsymbol{M}}_{\boldsymbol{x}}\left( T=0 \right)}\vert\boldsymbol{x} \right]$  =$\int E[Y\vert T=1,\boldsymbol{M},\boldsymbol{x}]dF(\boldsymbol{M}\vert T=1,\boldsymbol{x})-\int E[Y\vert T=1,\boldsymbol{M},\boldsymbol{x}]dF(\boldsymbol{M}\vert T=0,\boldsymbol{x})$  $\mathrm{DE}=E\left[ Y_{{T=1, \boldsymbol{M}}_{\boldsymbol{x}}\left( T=0 \right)}-Y_{{T=0, \boldsymbol{M}}_{\boldsymbol{x}}\left( T=0 \right)}\vert\boldsymbol{x} \right]$  $=\int\left\{ E\left[ Y \vert T=1,\boldsymbol{M},\boldsymbol{x} \right]- E\left[ Y \vert T=0,\boldsymbol{M},\boldsymbol{x} \right] \right\}dF\left( \boldsymbol{M} \vert T=0,\boldsymbol{x} \right)$ |

Derivation*: the completed derivations for MDM and RDM expressions are provided in Section S1. The completed derivations for ME and DE expressions can be found in Section S2 in SparseMCMM [9].

**Section S6 SparseMCMM_HD web app instruction**

The SparseMCMM_HD web app (<https://chanw0.shinyapps.io/sparsemcmm_hd/>) has four panels: PSM, Association analysis, Health disparity analysis, and Workflow. The Workflow panel, as shown in Figure S11, summarizes what our proposed SparseMCMM_HD framework is and introduces three analyzing steps: propensity score matching (PSM) analysis, group comparison regarding the outcome and microbial community diversity, and health disparity measures estimation and testing, whose results are correspondingly displayed in PSM, Association analysis, and Health disparity analysis three panels, respectively.

This interactive web app offers users a simple and engaging approach to analyze the microbiome data. As left panel shows (Figure S12), all that users need to input or choose are: the dataset to be analyzed; whether or not PSM is utilized; if PSM is required, the matching covariates and the exact matching covariates to be used (NULL if no exact matching covariate is needed); the exposure of interest; the reference exposure group; the outcome of interest; the taxonomic rank for health disparity analysis (we recommend Family, Genus, or Species); and number of split used for post-selection inference. If the number of splits is greater than one, we report both the mean and the 95% confidence interval estimates for health disparities based on these splits. If there is only a single split, we report the point estimates instead.

Here we take the China-USA comparison from curatedMetagenomicDataV3.4.2 R package discussed in the main text as an example for illustration. 570 samples from China and 350 samples from the USA were used in the China-USA comparison. PSM was performed to control for age and gender, with gender being used for exact matching. The PSM panel displays histogram plots of propensity scores before and after matching in the reference and comparison groups (Figure S13). The Association analysis panel in Figure S14 shows the results of group comparisons based on the matched dataset provided by the PSM procedure. These results correspond to our analysis discussed in the main text, including group comparison results based on t-test for the outcome and alpha diversities, multivariate analysis of variance (PERMANOVA) test for beta diversities, and their visualizations. The Health disparity analysis panel in Figure S15 displays estimates of ODM, RDM, and MDM, p-values of two test statistics OMD and CMD based on 500 permutations, and point estimates of component-wise MDMs for the identified species that have mediation effects based on one data split.


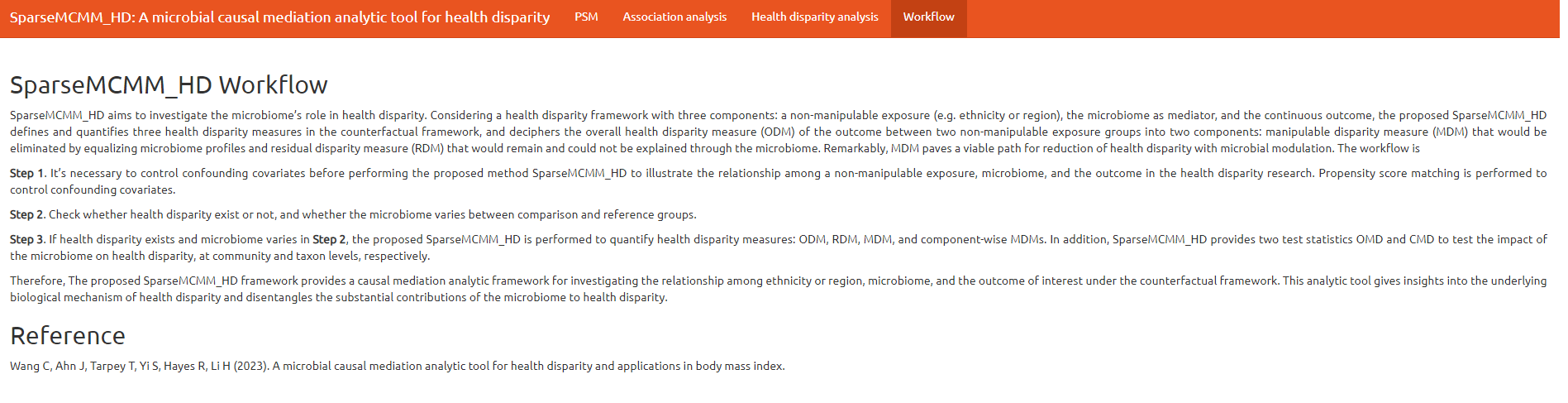


**Figure S11**. The Workflow panel in the SparseMCMM_HD web app.


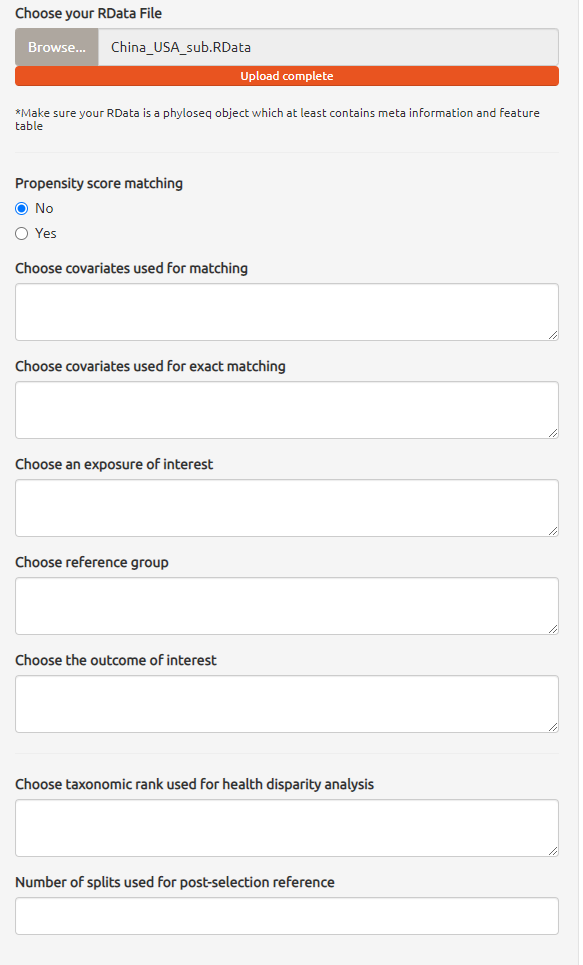


**Figure S12**. The left panel of the SparseMCMM_HD web app.


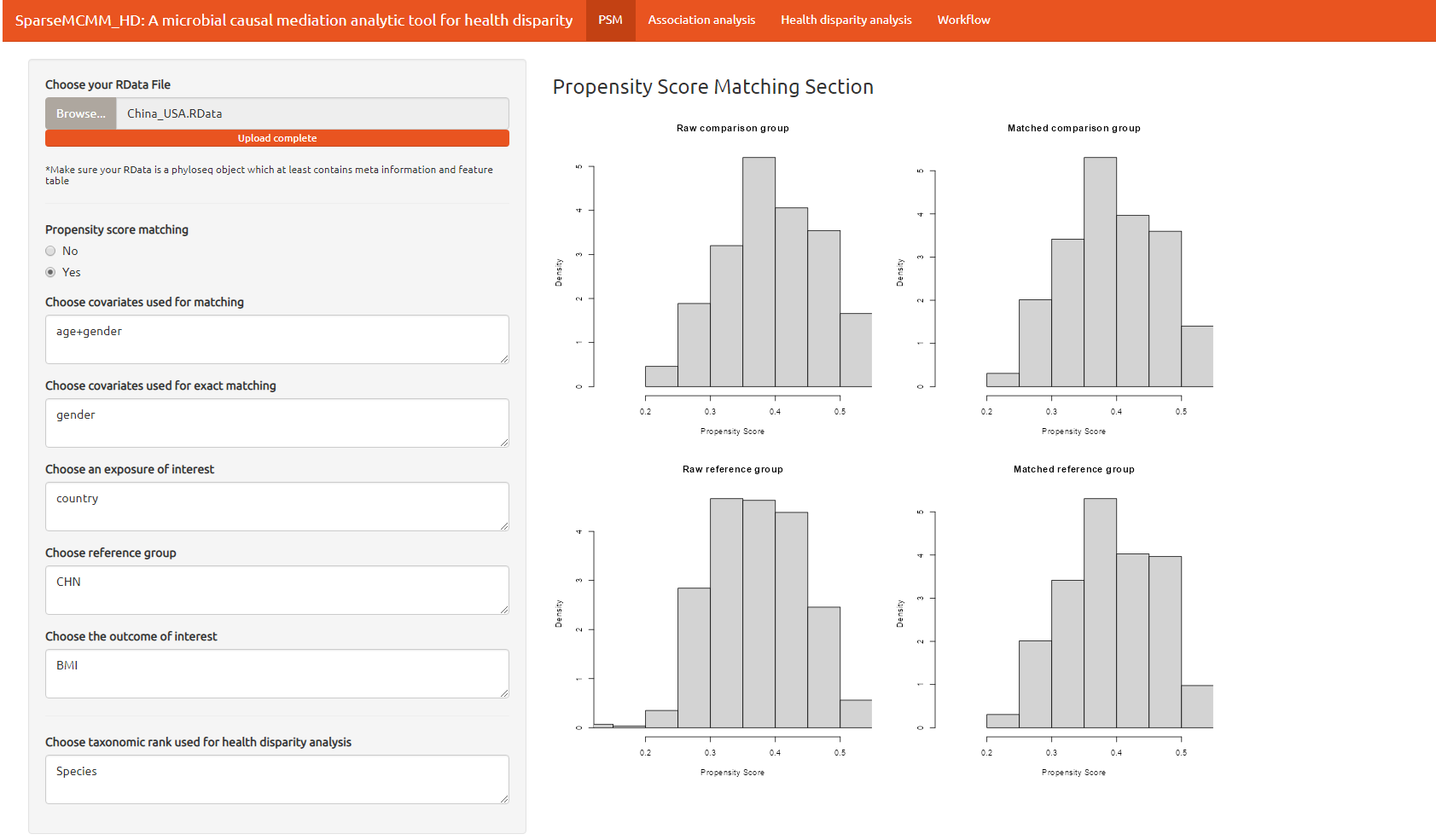


**Figure S13**. PSM results displayed in the PSM panel in China-USA comparison analysis.


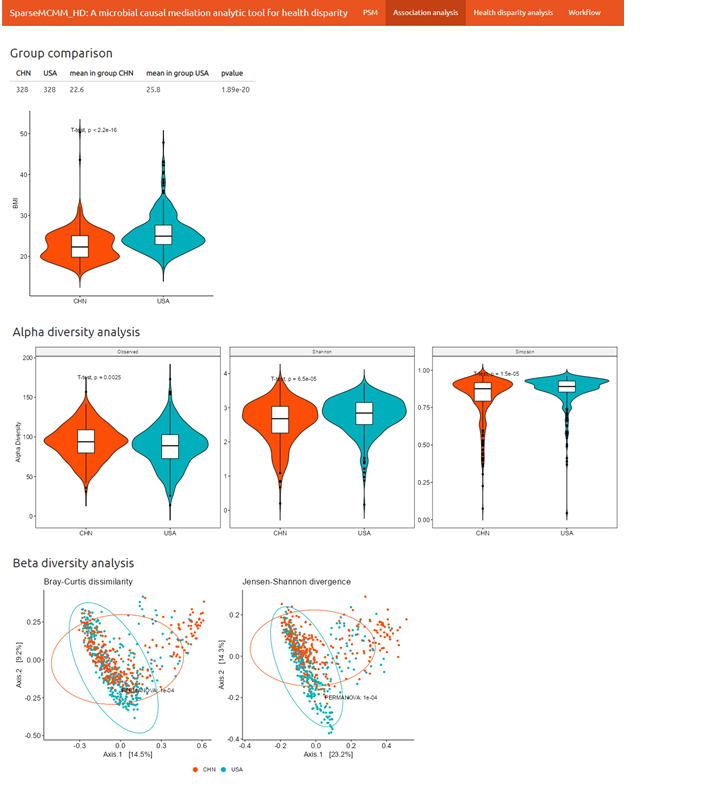


**Figure S14**. Group comparison results displayed in the Association analysis panel in the matched China-USA dataset in China-USA comparison analysis.


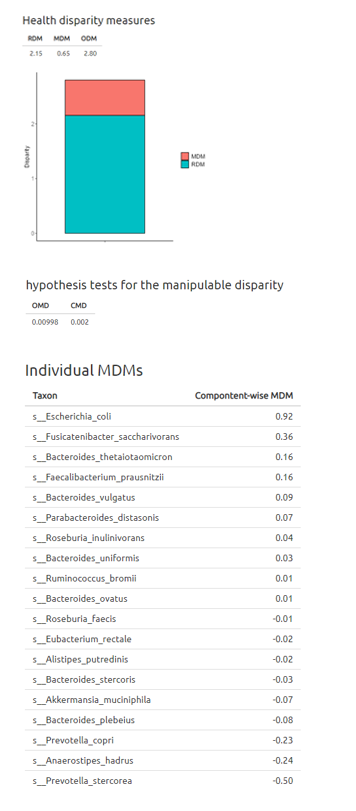


**Figure S15**. Results displayed in the Health disparity analysis panel in the matched China-USA dataset in China-USA comparison analysis.

**References**

1. VanderWeele, T.J. and W.R. Robinson, *On causal interpretation of race in regressions adjusting for confounding and mediating variables.* Epidemiology (Cambridge, Mass.), 2014. **25**(4): p. 473.

2. Naimi, A.I., et al., *Mediation analysis for health disparities research.* American journal of epidemiology, 2016. **184**(4): p. 315-324.

3. McDonald, D., et al., *American gut: an open platform for citizen science microbiome research.* Msystems, 2018. **3**(3): p. e00031-18.

4. Hu, J., et al., *A two-stage microbial association mapping framework with advanced FDR control.* Microbiome, 2018. **6**(1): p. 1-16.

5. Liu, M., et al., *Oxalobacter formigenes-associated host features and microbial community structures examined using the American Gut Project.* Microbiome, 2017. **5**(1): p. 1-17.

6. Rosenbaum, P.R. and D.B. Rubin, *The central role of the propensity score in observational studies for causal effects.* Biometrika, 1983. **70**(1): p. 41-55.

7. Austin, P.C., *An introduction to propensity score methods for reducing the effects of confounding in observational studies.* Multivariate behavioral research, 2011. **46**(3): p. 399-424.

8. McMurdie, P.J. and S. Holmes, *phyloseq: an R package for reproducible interactive analysis and graphics of microbiome census data.* PloS one, 2013. **8**(4): p. e61217.

9. Wang, C., et al., *Estimating and testing the microbial causal mediation effect with high-dimensional and compositional microbiome data.* Bioinformatics, 2020. **36**(2): p. 347-355.

10. Sohn, M.B. and H. Li, *Compositional mediation analysis for microbiome studies.* The Annals of Applied Statistics, 2019. **13**(1): p. 661-681.

11. Zhang, J., Z. Wei, and J. Chen, *A distance-based approach for testing the mediation effect of the human microbiome.* Bioinformatics, 2018. **34**(11): p. 1875-1883.

12. Zhang, H., et al., *Mediation effect selection in high‐dimensional and compositional microbiome data.* Statistics in medicine, 2021. **40**(4): p. 885-896.

13. Yue, Y. and Y. Hu, *Testing Mediation Effects in High-Dimensional Microbiome Data with False Discovery Rate Control.* 2021.

14. Wu, Q., et al., *MarZIC: A Marginal Mediation Model for Zero-Inflated Compositional Mediators with Applications to Microbiome Data.* Genes, 2022. **13**(6): p. 1049.
