# Supplementary material for: A microbial causal mediation analytic tool for health disparity and applications in body mass index
Source: Microbiome. 2023 Jul 27;11:164. doi: 10.1186/s40168-023-01608-9 (PMC10373330; doi:10.1186/s40168-023-01608-9)
Supplement: Supplementary file 3 — Additional file 2: Figure S1. Flowcharts for data pre-processing in the AGP dataset. a Pre-processing for all covariates. b The sample breakdown for the disparity analysis. Figure S2. Plots of standardized mean differences before and after propensity score matching for the datasets from the curatedMetagenomicData package [28]. a Comparison between Chinese and USA subjects. b Comparison between Chinese and UK subjects. Figure S3. Histogram plots of propensity score before and after propensity score matching for the datasets from the curatedMetagenomicData package [28]. a Comparison between Chinese and USA subjects. b Comparison between Chinese and UK subjects. Figure S4. Glycine lipid biosynthesis pathway generated based on MetaCyc database (https://metacyc.org/). The gene from B.thetaiotaomicro is located in an operon together with a second gene, glsA, which encodes the second enzyme of the pathway, an O-acyltransferase that forms the diacylated compound. Figure S5. The species with opposite mediation directions in the disparity of BMI between China-USA and China-UK comparisons. a Violin plots illustrating the relative abundances of these identified species in the matched Chinese and USA samples, and the matched Chinese and UK samples, respectively. b Scatterplots of BMI and the relative abundances of these identified species in the matched Chinese and USA samples, and the matched Chinese and UK samples, respectively. Figure S6. The species playing mediation roles in the disparity of BMI in the comparison between Chinese and USA subjects only. a Violin plots illustrating the relative abundances of these identified species in the matched Chinese and USA samples. b Scatterplots of BMI and the relative abundances of these identified species in the matched Chinese and USA samples. Figure S7. The species playing mediating roles in the disparity of BMI in the comparison between Chinese and UK subjects only. a Violin plots illustrating the relative abundances of these iden [file 40168_2023_1608_MOESM2_ESM.docx]

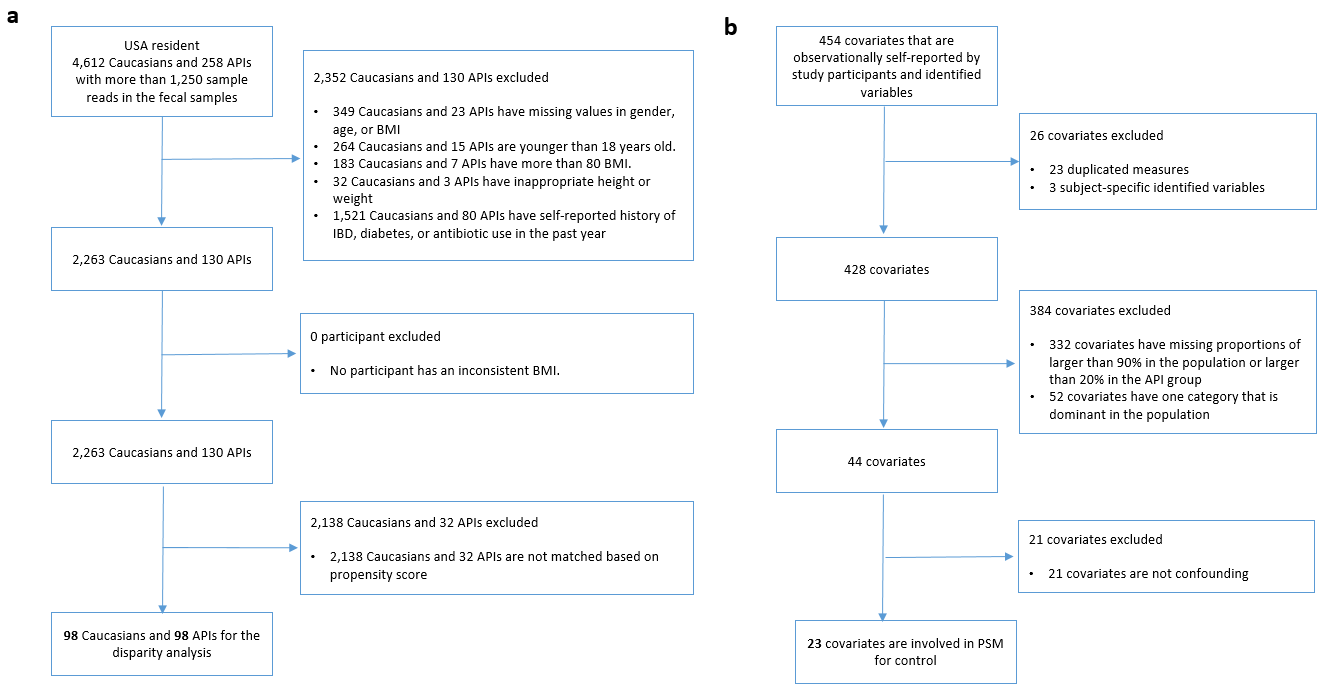


**Figure S1.** Flowcharts for data pre-processing in the AGP dataset. **a** Pre-processing for all covariates. **b** The sample breakdown for the disparity analysis.


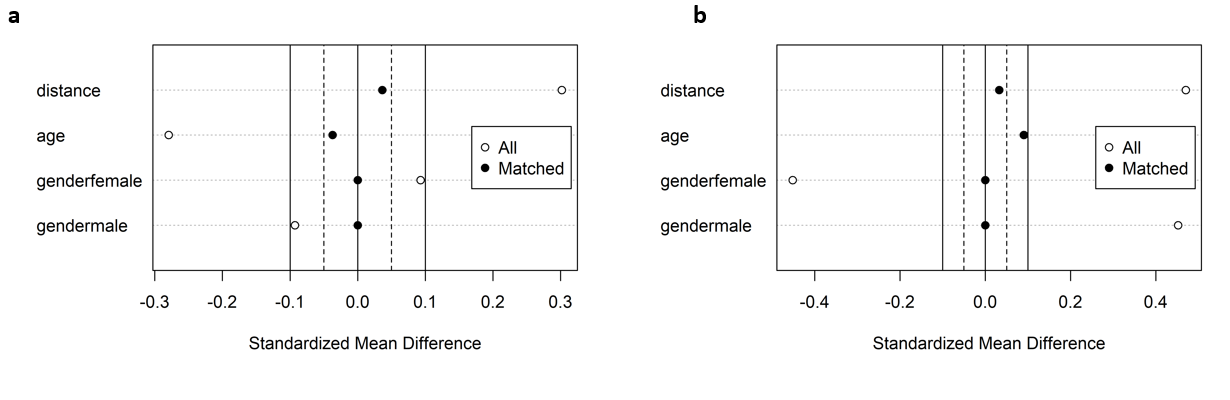


**Figure S2.** Plots of standardized mean differences before and after propensity score matching for the datasets from the curatedMetagenomicData package [1]. **a** Comparison between Chinese and USA subjects. **b** Comparison between Chinese and UK subjects.


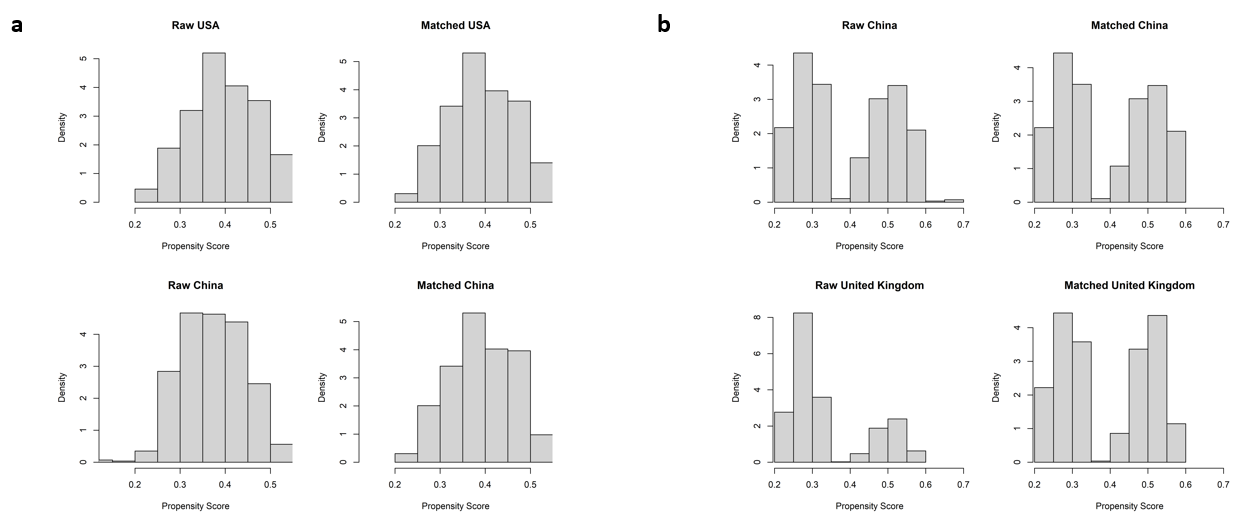


**Figure S3.** Histogram plots of propensity score before and after propensity score matching for the datasets from the curatedMetagenomicData package [1]. **a** Comparison between Chinese and USA subjects. **b** Comparison between Chinese and UK subjects.


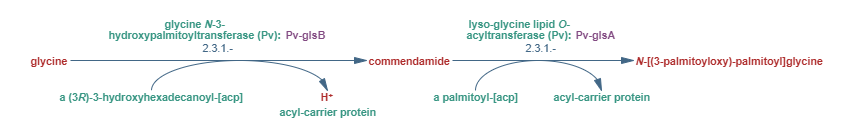


**Figure S4.** Glycine lipid biosynthesis pathway generated based on MetaCyc database (<https://metacyc.org/>). The gene from *B.thetaiotaomicro* is located in an operon together with a second gene, glsA, which encodes the second enzyme of the pathway, an *O*-acyltransferase that forms the diacylated compound.


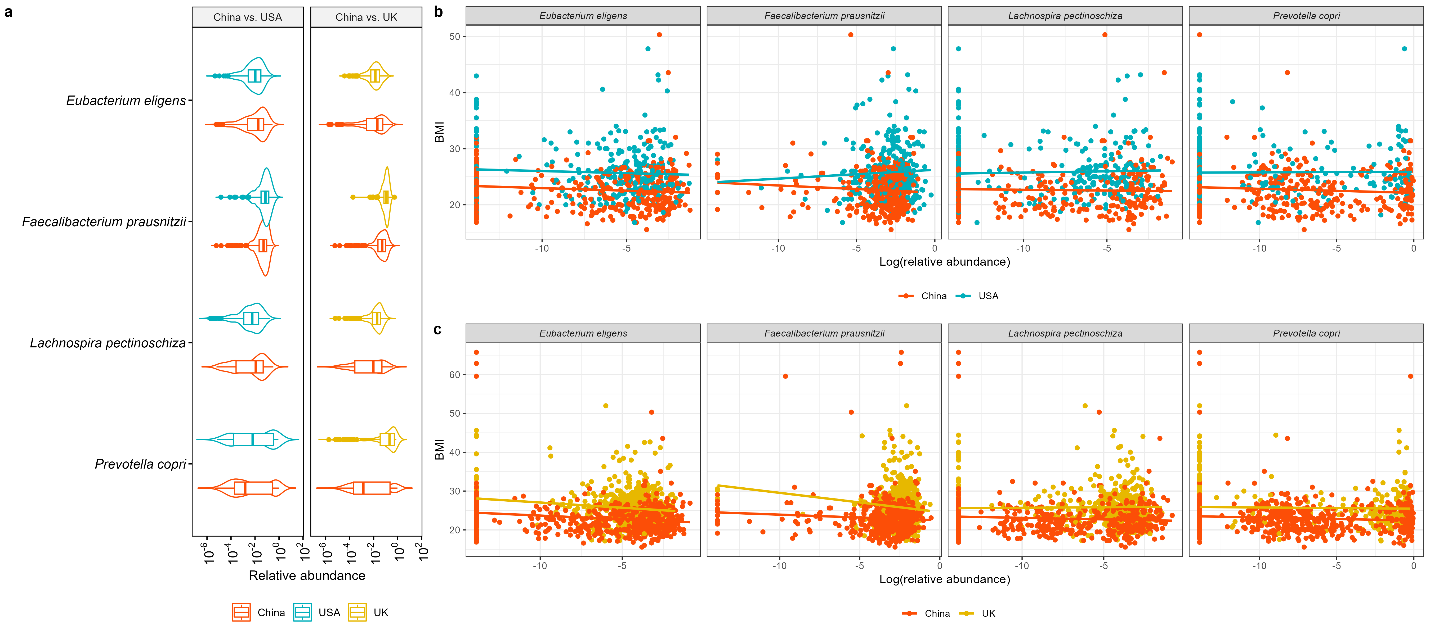


**Figure S5.** The species with opposite mediation directions in the disparity of BMI between China-USA and China-UK comparisons. **a** Violin plots illustrating the relative abundances of these identified species in the matched Chinese and USA samples, and the matched Chinese and UK samples, respectively. **b** Scatterplots of BMI and the relative abundances of these identified species in the matched Chinese and USA samples, and the matched Chinese and UK samples, respectively.


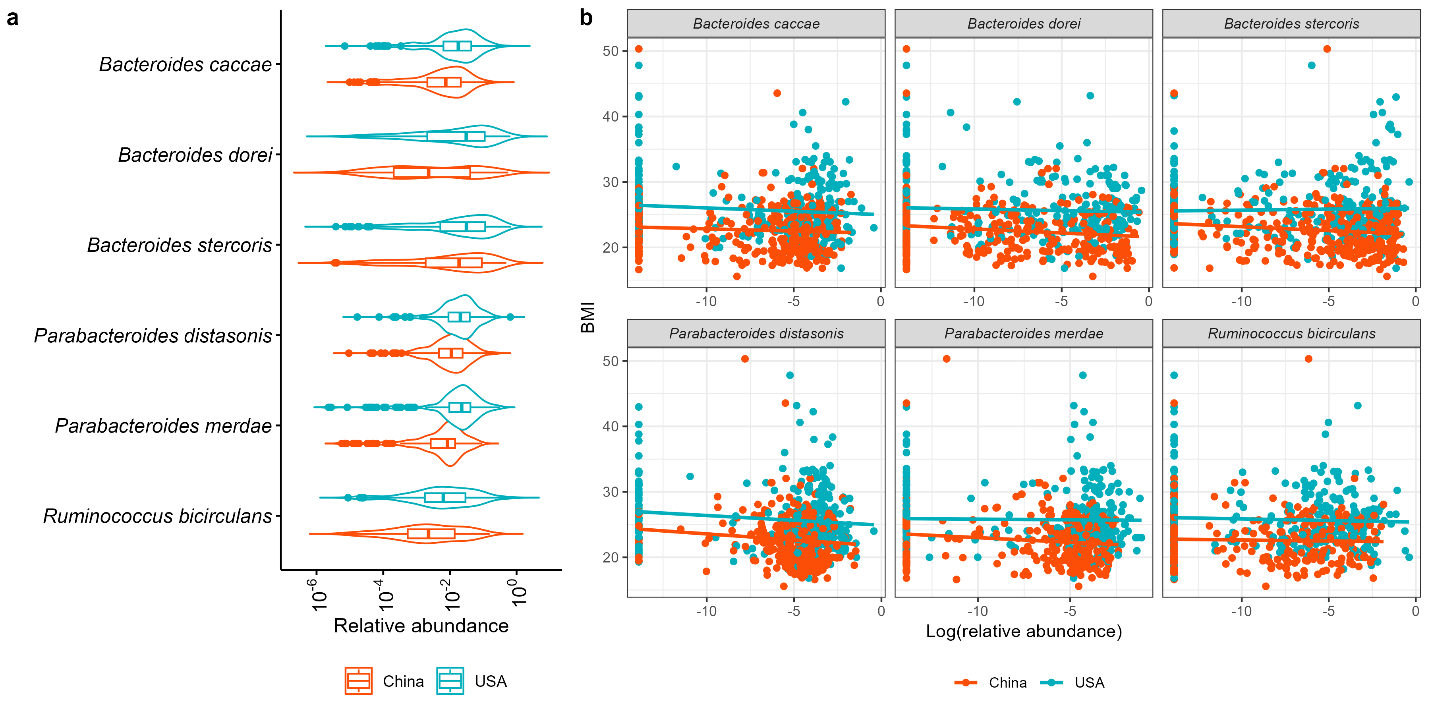


**Figure S6.** The species playing mediation roles in the disparity of BMI in the comparison between Chinese and USA subjects only. **a** Violin plots illustrating the relative abundances of these identified species in the matched Chinese and USA samples. **b** Scatterplots of BMI and the relative abundances of these identified species in the matched Chinese and USA samples.


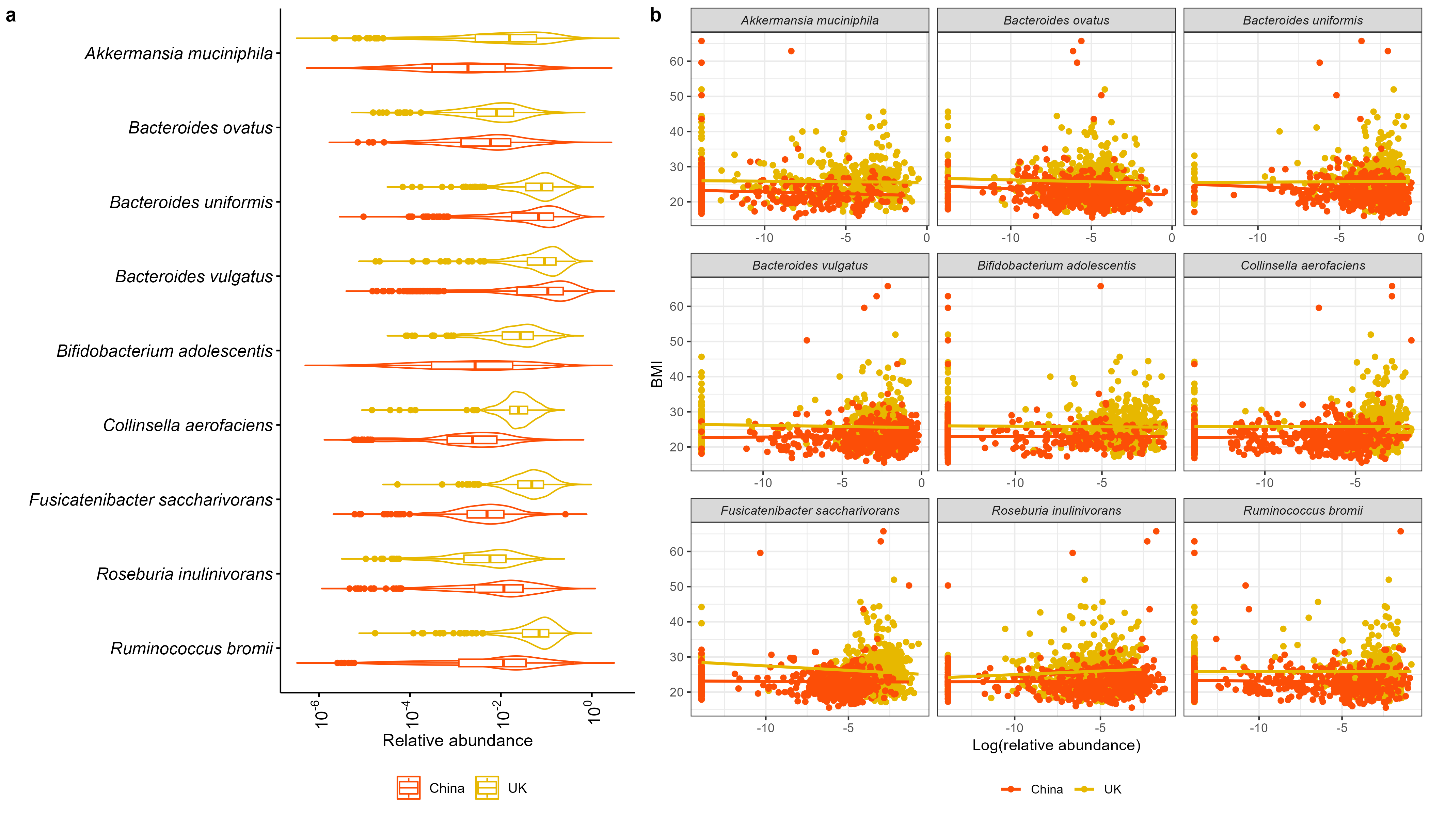


**Figure S7.** The species playing mediating roles in the disparity of BMI in the comparison between Chinese and UK subjects only. **a** Violin plots illustrating the relative abundances of these identified species in the matched Chinese and UK samples. **b** Scatterplots of BMI and the relative abundances of these identified species in the matched Chinese and UK samples.


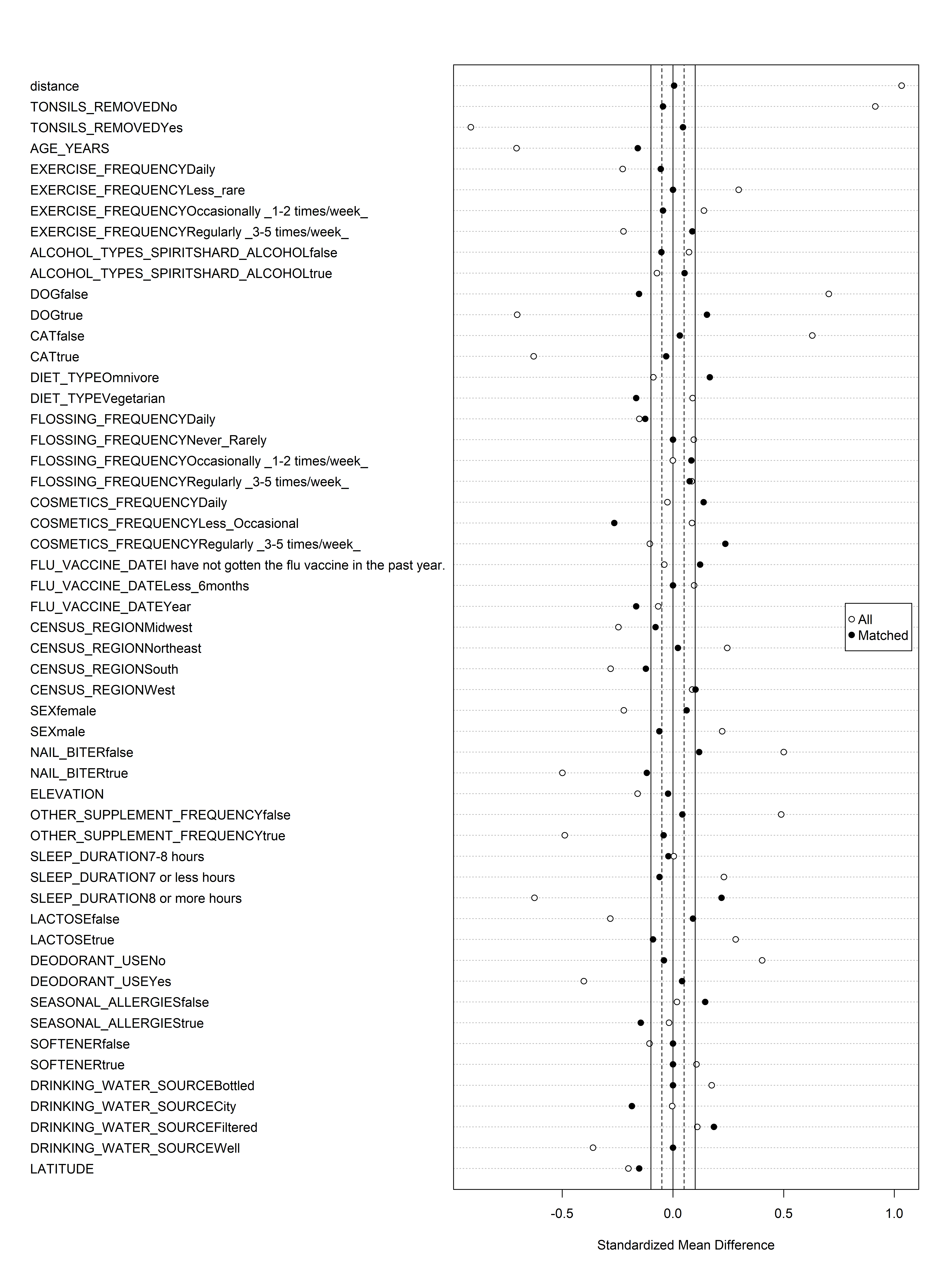


**Figure S8.** Plots of standardized mean differences before and after propensity score matching for the comparison between the API and Caucasian samples from the AGP dataset. API: Asian or Pacific Islander.

**
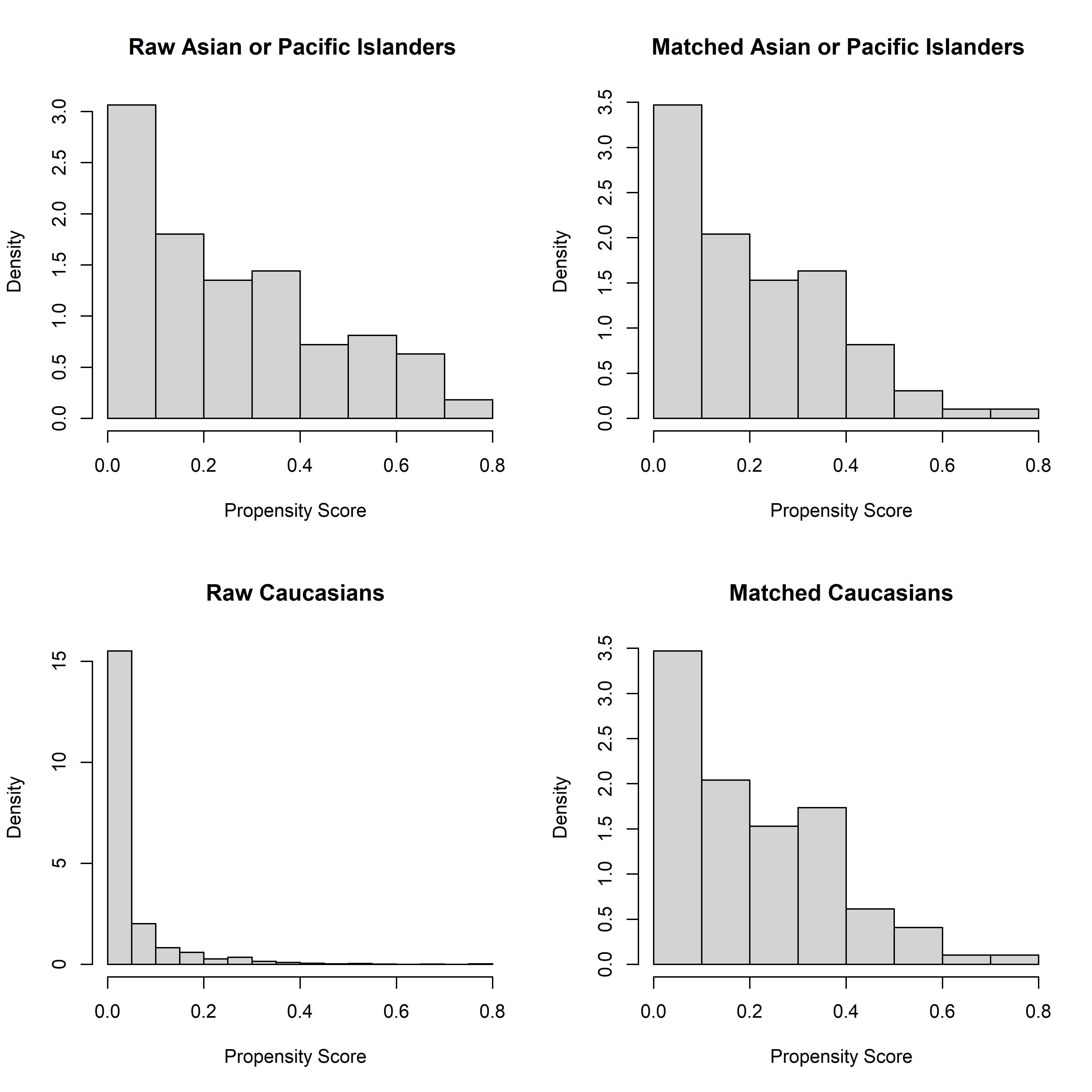
**

**Figure S9.** Histogram plots of propensity score before and after propensity score matching for the comparison between the API and Caucasian samples from the AGP dataset. API: Asian or Pacific Islander.

**
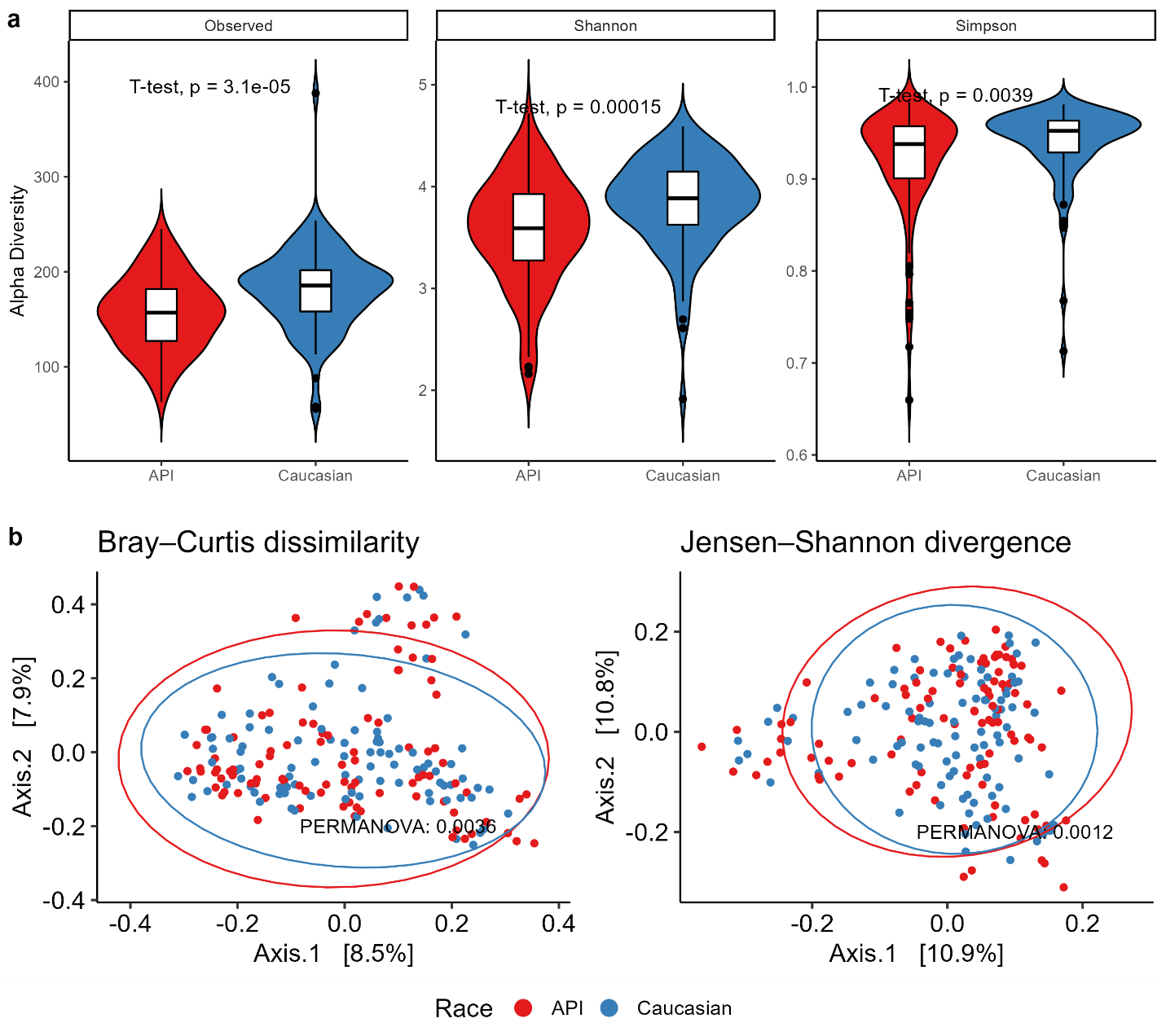
**

**Figure S10.** Association analyses in the AGP dataset. **a** Violin plots of alpha diversities including Observed, Shannon, and Simpson indices in the matched API and Caucasian samples. **b** PCoA plots using Bray–Curtis dissimilarity and Jensen–Shannon divergence in the matched API and Caucasian samples. API: Asian or Pacific Islander.

**Reference**

1. Pasolli, E., et al., *Accessible, curated metagenomic data through ExperimentHub.* Nature methods, 2017. **14**(11): p. 1023-1024.
